# Organic and Metal–Organic Polymer-Based Catalysts—Enfant Terrible Companions or Good Assistants? †

**DOI:** 10.3390/molecules29194623

**Published:** 2024-09-29

**Authors:** Milan Králik, Peter Koóš, Martin Markovič, Pavol Lopatka

**Affiliations:** Institute of Organic Chemistry, Catalysis and Petrochemistry, Slovak University of Technology, Radlinského 9, 812 37 Bratislava, Slovakia; martin.markovic@stuba.sk (M.M.); pavol.lopatka@stuba.sk (P.L.)

**Keywords:** organic polymer, resin, metal–organic framework, catalyst, metal, preparations, characterization, deactivation

## Abstract

This overview provides insights into organic and metal–organic polymer (OMOP) catalysts aimed at processes carried out in the liquid phase. Various types of polymers are discussed, including vinyl (various functional poly(styrene-co-divinylbenzene) and perfluorinated functionalized hydrocarbons, e.g., Nafion), condensation (polyesters, -amides, -anilines, -imides), and additional (polyurethanes, and polyureas, polybenzimidazoles, polyporphyrins), prepared from organometal monomers. Covalent organic frameworks (COFs), metal–organic frameworks (MOFs), and their composites represent a significant class of OMOP catalysts. Following this, the preparation, characterization, and application of dispersed metal catalysts are discussed. Key catalytic processes such as alkylation—used in large-scale applications like the production of alkyl-*tert*-butyl ether and bisphenol A—as well as reduction, oxidation, and other reactions, are highlighted. The versatile properties of COFs and MOFs, including well-defined nanometer-scale pores, large surface areas, and excellent chemisorption capabilities, make them highly promising for chemical, electrochemical, and photocatalytic applications. Particular emphasis is placed on their potential for CO_2_ treatment. However, a notable drawback of COF- and MOF-based catalysts is their relatively low stability in both alkaline and acidic environments, as well as their high cost. A special part is devoted to deactivation and the disposal of the used/deactivated catalysts, emphasizing the importance of separating heavy metals from catalysts. The conclusion provides guidance on selecting and developing OMOP-based catalysts.

## 1. Introduction

The term “catalysis” is widely used both in technical practices and social processes. The chemical compound that accelerates a process is referred to as a catalyst. In natural sciences and technical practice, a catalyst is a specific chemical or electromagnetic substance that participates in a chemical reaction and increases the reaction rate [1,2]. More than 90% of industrial chemical processes are catalytic, and virtually all transformation processes in living organisms are catalyzed with peculiar substances such as enzymes and hormones [3,4,5]. These biochemical transformations typically occur at relatively low temperatures (e.g., in the human body), under atmospheric pressure, and mainly involve stereoselective reaction routes, which allow the production of products with necessary stereospecific properties.

The effect of catalysts has been recognized since ancient times. Aristotle (384–322 BC) already specified active substances (catalysts) and passive substances (reactants), that undergo transformation [6]. In 1835, Berzelius [7] proposed the following definition: “A catalyst is any substance including light, that directly alters the rate of a chemical reaction without entering into the net chemical reaction itself”. According to this definition, a catalyst can either increase or decrease/retard the reaction rate. In modern catalysis, only substances that increase the reaction rate are considered catalysts, while substances that lower the rate are referred to as inhibitors. Inhibitors are widely used to slow down the oxidation or polymerization process [8]. In 1896, Ostwald [6,7] provided a more precise definition that “a catalyst is a substance, which accelerates a chemical reaction, and it is not consumed in the course of this reaction”. A definition with the same meaning is stated by IUPAC [9]. This definition, as well as the explanation of catalysis found in textbooks (see, e.g., [1,10,11]), describes the action of a catalyst as lowering the energetic barriers required for the activation of reactants. Surprisingly, discussions about negative catalysts still persist [12]. It is worth noting that inhibitors were defined more than 100 years ago (1923) [13], and in 1969, they were discussed in relation to activation energy [14] as follows: “it is difficult to think of a mechanism by which a negative catalyst can provide an alternate path to a reaction with a higher energy of activation”.

The catalytic processes can be broadly categorized into the following two basic types based on the form of the catalyst and its “miscibility” with the reaction mixture:

- Homogeneous catalysis (the catalyst and reactants are in the same phase).
- Heterogeneous catalysis (the catalyst (solid, liquid) and reactants/products are in different phases).

The advantage of *homogeneous catalysis* lies in its high catalytic performance due to the excellent accessibility of catalytic centers by the reactants, resulting in high catalytic activity. However, a disadvantage is the more complicated isolation of the catalyst from the reaction mixture. These features are inverted for heterogeneous catalysis, i.e., access to catalytic centers is hindered by external and internal mass transports, but the catalyst is easily separated from the reaction mixture. The latter feature is particularly advantageous for reactors with a fixed bed catalyst, which are widely used in large-scale productions, e.g., petrochemical processes such as hydrogenation, hydrocracking, alkylation, etc. [15]. A noteworthy variation is *heterogenized catalysis*, where the positive attributes of homogeneous catalysis are leveraged by anchoring catalytic moieties to a solid support. For example, resin-based catalysts such as sulfonated polystyrene-co-divinylbenzene are utilized in alkylation reactions, e.g., the formation of alkyl *tert*-butyl ethers, which are important ecological additives in motor fuels [15]. Another approach involves hybridized *organocatalysts*, where moieties containing “catalytic” heteroatoms are embedded within a polymer framework [10,16].

Catalytic processes can be further classified depending on the additional energies applied to the reaction system, particularly mechanical or electromagnetic irradiation. A finer division of catalytic processes includes the following [6,10]:

i. Microwave catalysis [17] and sonocatalytic processes [18,19,20].
ii. Mechanocatalysis [21,22,23].
iii. Magnetodriven catalysis [24].
iv. Photocatalysis, including light-driven processes [25,26].
v. Plasmonic catalysis [27,28,29].
vi. Piezocatalysis, piezoelectrocatalysis [30,31,32], and other processes.

A special category belongs to electrocatalysis [5,33,34,35], in which the rearrangement of electrons in the valence shells of reactants proceeds via reduction and oxidation steps at the cathode and anode, respectively. Effective electrocatalysts can reduce the potential difference between electrodes and increase current efficiency (also called Faraday’s efficiency) [36,37]. This allows electrodes to operate at lower temperatures and reduces the amount of electric energy converted to heat, thus improving overall current efficiency.

Catalysts based on organic and metal–organic polymers (OMOPs) are relatively new compared with inorganic-based ones, which arose from the later development and application of organic polymers, starting around the mid-20th century. For instance, in one of the earliest catalytic books from 1940 by Berkman et al., only “wood” and wool were mentioned as supports for metal species [38]. Initially, organic functional polymers were primarily used as acid catalysts [39,40]. Later, metal dispersed, and multifunctional catalysts were developed [41,42]. The potential for diverse texture (e.g., porosity and pore distribution), elasticity (which reduces the resistance of mass transport compared to inorganic rigid catalysts), and hydrophilic/hydrophobic properties and the possibility of functionalization led to the belief of super-properties of such catalysts. However, it later became apparent that the lifetime of such catalysts, particularly mechanical and chemical stability, significantly decreases at higher temperatures. Such properties and reduction in stability can cause technological, economic, and ecological problems. In this post, we will try to indicate the fine line between these catalysts behaving as “infant terrible companions” and serving as “good assistants”.

## 2. Catalysts—Kinetics, Mass, and Heat Transport

The next section will focus on heterogeneous catalysis. The catalytic centers of solid heterogeneous catalysts include the following four types:

- Properties of the solid material, including features such as the concentration of Bronsted and Lewis acid sites, e.g., in the case of zeolites, defects in oxide supports, and heteroatoms in a polymer network (organocatalysis).
- Anchored, well-defined functional groups, e.g., sulfonic, amino, tetraammonium, carboxylic, metal–organic, and similar groups.
- Catalytic particles, represented mainly by metal nanoparticles, oxides, and carbides.
- Single atoms fixed within a network of polymer support, falling under the category of single-atom catalysis (SAC).

The catalytic activity of solid catalytic particles is strongly dependent on their size, particularly in the range below 10 nm [5,43,44]. A well-documented example of this dependence is how decreasing the size of bulk gold metal, typically chemically inactive, can transform it into a superficial catalyst, as described in many studies [45,46,47,48,49].

**Figure 1:**
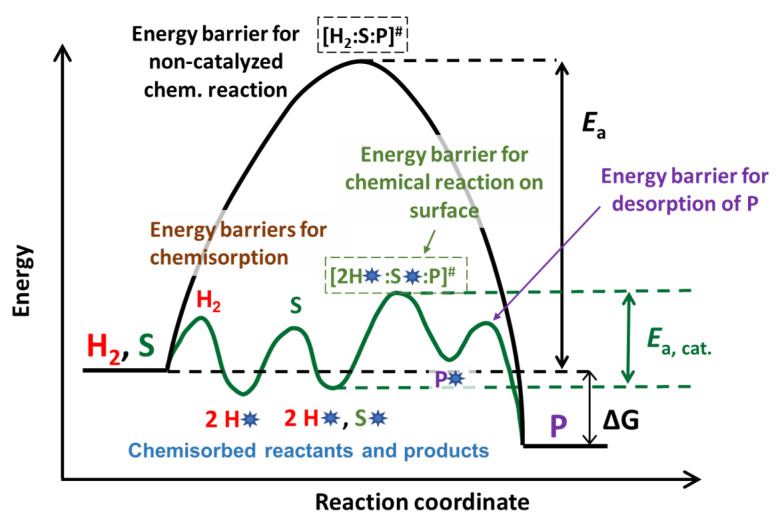
The energy profile of catalyzed and non-catalyzed hydrogenation.

To elucidate a catalytic process, the hydrogenation of a substrate **S** was chosen. Figure 1 depicts the energy changes in the reaction system, which include the following steps:

- Chemisorption and dissociation of hydrogen:

**H_2_** + 2 * ⇄ 2 H*;

where * denotes a catalytic center and H* represents an activated (chemisorbed) moiety.

- Chemisorption of the substrate:

**S** + * ⇄ S*.

- Surface reaction:

S* + H* ⇄ SH* + *.

SH* + H* ⇄ P* + *.

- Desorption of the product:

P* ⇄ **P** + *.

The term [2H*:S*:**P**]^#^ in Figure 1 represents an intermediate in the catalytic hydrogenation process. For comparison, a non-catalytic reaction coordinate includes an intermediate [**H_2_**:**S:P**]^#^. The essence of “catalysis“ lies in overcoming a much lower energy barrier (***E*_a,cat_**_._) compared with a non-catalyzed reaction (***E*_a_**). From a kinetic perspective, the reaction rate is typically determined by the slowest step, which usually involves the highest energy barrier [5,50]. The rate-determining step of the process could be one of the following:

i. Chemisorption of one or both reactants.
ii. Surface reaction.
iii. Desorption of a product.

According to the plot in Figure 1, the rate-determining step in this case is the surface reaction involving chemisorbed reaction intermediates. 

It is important to note that non-catalytic hydrogenation reactions with hydrogen typically occur only at higher temperatures, approximately above 500 °C, and are often accompanied by a series of more complex reactions, e.g., hydrogenolysis and cracking. Non-catalytic hydrogenations at lower temperatures can proceed when hydrogen is transferred from/through a donor molecule, as seen in the non-catalytic liquefaction of coal [51]. Figure 1 illustrates the most commonly occurring heterogeneous catalytic process, modeled by the reaction of chemisorbed reactants. These models are known as Langmuir–Hinshelwood–Houghen–Watson (LHHW) models, sometimes named in abbreviated form as Langmuir–Hinshelwood (LH) models [1,50]. Another type of catalytic process, in which one of the reactants is chemisorbed on a catalytic surface and another reactant attacks the chemisorbed reactant, is the Elley–Rideal model [11]. A distinct group of catalytic processes is represented by Mars–van Krevelen redox models. In these processes, a reactant, such as oxygen, is incorporated into the catalyst’s structure, and the incorporated species (chemically bound) subsequently reacts with another reactant [11]. Examples of the latter processes include the oxidation of SO_2_ to SO_3_, benzene to maleinanhydride, naphthalene to phthalanhydride, etc. [11,15], typically catalyzed by transition metal oxides, often vanadium. The Mars–van Krevelen (MvK) mechanism is characterized by changes in the valence state of the transition metals used, such as when the average oxidation state of vanadium changes between 4 and 5. It is necessary to add that the MvK mechanism occurs at temperatures higher than 200 °C, and for OMOP-type catalysts, it is not described in the available literature. LH and ER mechanisms govern catalysis with OMOPs [11,50]. 

**Figure 2:**
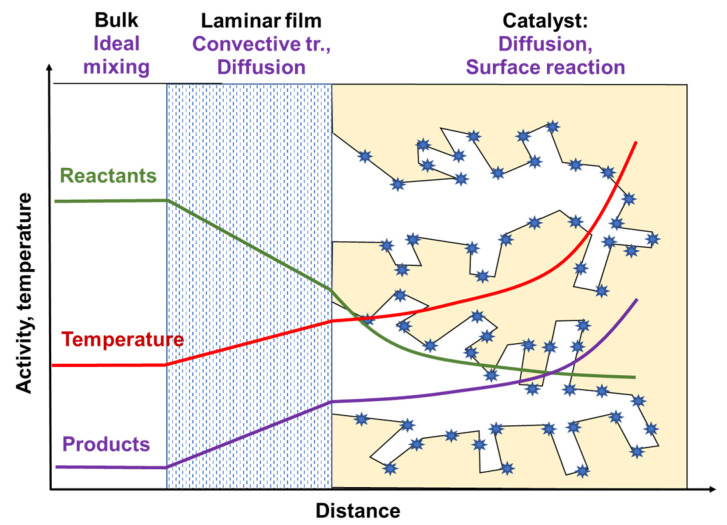
Mass transport and chemical reaction in an exothermic heterogeneously catalyzed process.

The discussed mechanisms and relevant models are applicable/valid in the so-called kinetic regime, i.e., mass transport does not hinder the catalytic process [11,50]. Catalytic processes that proceed under the kinetic regime are usually homogeneous. However, under certain conditions, where mass transport does not significantly hinder the catalytic process, heterogenous catalytic processes can also approach the kinetic regime. Figure 2 illustrates the following mass transport and reaction steps in a chemical process catalyzed by a solid catalyst:

i. (External) mass transport—diffusion of reactants from a bulk fluid through the layers of fluid embracing the catalysts to the catalyst surface.
ii. Intraparticle diffusion to catalytic centers (internal mass transport).
iii. Chemisorption of reactant/reactants.
iv. Surface reaction.
v. Desorption of products.
vi. Diffusion of products to the catalyst surface (internal mass transport).
vii. (External) mass transport of products through the fluid layers embracing the catalyst surface to bulk fluid.

For a bimolecular reaction such as hydrogenation, the energy profile for steps iii–v is depicted in Figure 1.

The fluid involved in catalytic processes can be either gas or liquid. Layers of fluid around the catalyst can be virtually stagnant (forming a laminar film) or partially mixed. For simplicity, a film with some thickness and resistance to mass transport is considered. Once the reactants reach the catalyst surface, they must access catalytic centers (denoted as asterisks) located near the surface and/or deeper in the catalyst body. The rate of transport is driven by activity gradients and is slowed down by low diffusion coefficients, which are influenced by the bulkiness of molecules (molar weight and shape). The topology of the catalyst, as outlined in Figure 2, assumes an ideal texture with macropores (>50 nm, transport is not hindered by pore walls), mesopores (>2, <50 nm, a mild influence of pore walls on the transport is observed), and micropores (<2 nm, a strong hindrance is present). The classification of pore types by their sizes is well-defined in the literature [52]. In rigid materials, transport diffusion in micropores is known as Knudsen diffusion [50,53], whereas in swollen gel-type materials, molecule movement is similar to that of a viscous medium [54,55,56,57,58].

The highest volume concentration of catalytic sites is typically found in micropores (<2 nm) because of their high specific surface. However, poor accessibility (resulting from low diffusion coefficients in pores smaller than 2 nm) can prevent the catalytic centers located in micropores from functioning effectively (at sufficient rate) in catalytic reactions. Therefore, materials with a hierarchical structure that include macro-, meso-, and micropores are considered best for catalytic purposes. For example, synthetic zeolites, which are commonly microporous in their original structure [59], can be synthesized or modified to incorporate a 3D structure containing both micropores as well as mesopores [60,61]. These materials have a wide range of applications, such as in chemical synthesis, treatment of natural resources [62,63], or pollutant degradation [64].

Macroporous catalysts, both inorganic and OMOP-based, are particularly effective in reactions involving bulky molecules, whether as reactants or products (including polymers). In these cases, microporosity is less critical, but the presence of active catalytic sites on the macropore wall is essential. A good example is the synthesis of polylactic acid, with a molar mass of approximately 110,000 g mol^−1^, using sulfonated, highly crosslinked poly(styrene-co-divinylbenzene) (SBA-15, specific surface area 45 m^2^ g⁻¹, acidity 4.7 mmol g⁻¹) doped with Lewis acids. This catalyst effectively demonstrates the potential of macroporous systems [65]. Despite polymerization being carried out at 150 °C, which exceeds the recommended operating temperature of 120 °C for SBA-15, the catalyst remains recyclable. It is also important to highlight the need for proper mixing to facilitate the transport of the product from the macropores of the catalyst.

In connecting the accessibility of catalytic sites on the surface (all types of pores), so-called dynamic interactions (movement of surface polymer nods) play a positive role, particularly when metal particles are applied [66,67]. This is a great advantage against solid (rigid) inorganic materials.

In terms of the temperature profile, an exothermic reaction causes a local increase in temperature, meaning the catalyst body can become hotter than its surface and surrounding bulk fluid. Because of this temperature gradient, heat is transported from the catalyst to the bulk fluid. The accumulation of heat within the body of a catalyst raises its temperature, which generally has a negative effect on the selectivity of organic reactions. This temperature increase is higher when the thermal conductivity of a catalyst and the reaction media is low, a situation typical for gas–solid reactions [11,50,68].

Controlling the temperature of catalysts at a certain value is crucial and requires dissipation of the reaction heat from the catalyst body to the bulk reaction mixture (exothermic reaction), while for an endothermic reaction, heat must be supplied. Additionally, if nanoscale solid catalytic particles are used, it is important to prevent their sintering and/or release into the reaction mixture. Beyond optimizing the size of solid catalysts, the concentration of catalytic centers is another important factor. This often leads to the use of supported catalysts, where the support can actively contribute to the catalytic performance or possess a passive role [5,41,50,69,70]. Passive support refers to a material that does not interact with the components of the reaction mixture, meaning it exhibits no chemisorption or interaction with the deposited catalytic particles. Typical passive supports are polyethylene, polypropylene, and others that do not contain functional groups, double bonds, or aromatic rings that could donate/share electrons. However, passive supports play a crucial role in stabilizing catalytic particles (such as metals or oxides) by trapping them within the polymer network, thereby significantly inhibiting their sintering. This stabilization helps maintain the dispersion and activity of catalytic particles over time.

Typical catalyst supports are inorganic such as activated carbon, metal oxides (including alumina and silica), solid carbonates, etc. [5,38,50]. Also, organic catalyst supports including natural and synthetic polymers are used [41,42,57,71,72,73,74].

To approach kinetic regime conditions, it is necessary to minimize external and internal mass transports. The intensive mixing of reaction components enables the minimization of external transport. In a gas-phase reaction, external transport hindrance can often be neglected. The effect of internal mass transport decreases with the decreasing size of catalyst particles. The Weisz–Prater criterion, which evaluates the ratio of the chemical reaction rate to the diffusion rate of reactants, is commonly applied [11,50,75]. However, it is necessary to note that very small catalytic particles pose challenges for their separation from the reaction mixture. In the case of fixed-bed catalytic reactors, hydraulic resistance increases with the decreasing size of catalytic particles. Generally, particles smaller than 50 µm are not suitable for batch applications (stirred reactors), and particles smaller than 2 mm are unsuitable for fixed-bed reactors. Catalysts for fixed-bed industrial reactors have a characteristic dimension of 5–10 mm and often feature special shapes, e.g., rings, cylinders with hollows, stars, and similar shapes [5,50].

A particular problem arises when estimating reaction rate constants. If it is not possible to “separate true reaction kinetics” and mass transport, the estimation of kinetic parameters is performed while accounting for reactant diffusion [11,75,76,77,78].

As illustrated by the facts above (previous paragraphs and the Introduction), catalysis represents a very complex process characterized by interactions at the picometric (10^−12^ m, interactions of electrons), nanometric (10^−9^ m), and submicrometric (smaller than 10^−6^ m, mass and heat transport) levels. Additionally, certain effects of electromagnetic irradiation, such as those occurring at femtometric (10^−15^ m) and attometric (10^–18^ m) time scales (e.g., lasers and photons particles), must be considered. This complexity lends a rather “alchemistic essence” to catalysis, and it is no surprise that even modern textbooks (e.g., [1]) acknowledge that experimental data often surpass theoretical understanding. Therefore, catalysis remains predominantly an experimental field of technology, despite the availability of extensive databases and artificial intelligence tools. Particularly, extensive experiments under optimal conditions (type of catalyst, reactor setup, temperature, pressure, loading, etc.) are necessary for the successful development of specific technological processes. In this context, flow reactors [79] and microreactors are particularly valuable tools [80,81], as they effectively minimize mass and heat transfer limitations within the reaction system. A sophisticated example of a such system is described in reference [82], where polymer spheres (approximately 3 nm in size) with internal cages decorated with Pt/Ag nanoparticles function as microreactors. The effectiveness of this system was demonstrated in the hydrogenation of 4-nitrophenol, achieving nearly 100% conversion, near-perfect selectivity, and easy catalyst recyclability.

The topics discussed above were not strictly divided based on a type of solid heterogeneous catalyst. The subsequent sections will focus more specifically on organic polymer-based catalysts, although comparisons with inorganic catalysts will be included where appropriate.

## 3. Preparation of Organic and Metal–Organic Polymer (OMOP)-Based Catalysts

**Figure 3:**
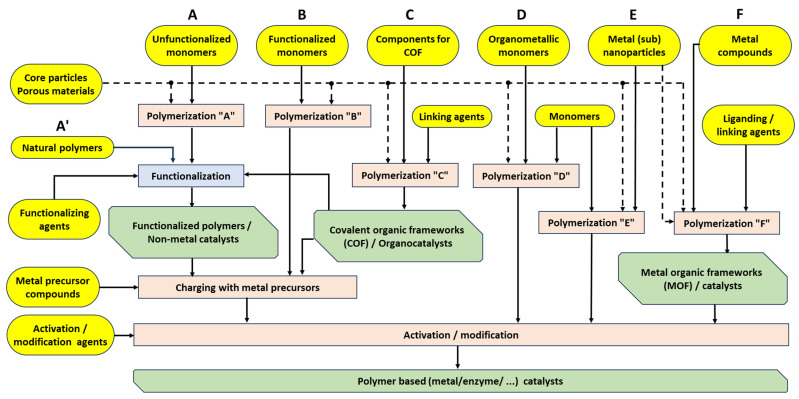
Preparation routes for organic, metal–organic, and enzyme polymer-based catalysts.

Based on data from the literature ([41,42,83,84,85,86,87,88,89,90,91,92,93,94,95,96,97,98,99]) and our experience ([54,55,76,100,101,102,103,104,105,106,107,108,109,110,111,112,113,114]), we prepared a comprehensive overview of the preparation of OMOP-based catalysts (Figure 3). The preparation routes are divided into two main groups as follows: **A**–**C**, which starts from pure organic monomers, and **D**–**F**, which involves metal compounds or even metal nanoparticles at the start (route **E**). The difficulty, complexity, and cost of the resulting catalysts increase from **A** to **D**. The characteristics of routes **E** and **F** depend on the type and cost of the starting materials. Although Figure 3 also includes the preparation of enzyme-heterogenized catalyst, this area will not be discussed further in this text, despite the availability of relevant published data [115,116,117].

OMOP-based polymers can be classified based on their origin (natural or synthetic), mechanical properties (rigid, plastic, or elastic), the type of chemical reaction used in the polymerization (addition or condensation), or the monomers used in the preparation of the desired polymer.

### 3.1. Vinyl Polymers

**Figure 4:**
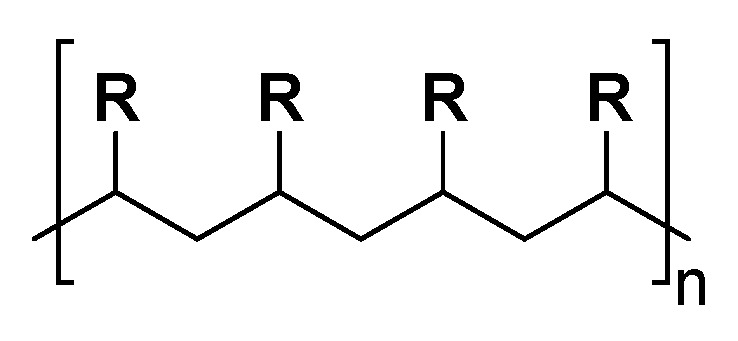
Vinyl-type polymers.

Vinyl monomers contain at least one double C=C bond, making them suitable for addition polymerization (Figure 4). Depending on the R substituent, the most common polymers are polyethylene (R = H, PE), polypropylene (R = CH_3_, PP), and polystyrene (R = C_6_H_5_, PS). Generally, if monomers contain only carbon and hydrogen atoms (unfunctionalized monomers), the resulting polymer is also unfunctionalized. However, when other elements, such as hetero atoms (O, N, P, S, etc.), are present in the monomer, the formed polymer is functionalized. Typical examples of such functionalized monomers are acrylic acid (R = COOH), vinyl acetate (R = O-COO-CH_3_) yielding polyvinyl alcohol (R = OH) after hydrolysis, acrylonitrile (R = CN), and amide-type monomers (R = CO-NH_2_ and/or R = CO-N(CH_3_)_2_).

If a polymer network formed by chemically bound species (crosslinked polymers) is required, monomers with at least two polymerizable groups are used. Examples of such monomers include butadiene (CH_2_=CH-CH=CH_2_), divinylbenzene (CH_2_=CH-C_6_H_4_-CH=CH_2_), and *N*,*N*’-methylenebisacrylamide (CH_2_=CH-CO-NH-CH_2_-NH-CO-CH=CH_2_). The degree of crosslinking can vary and depends on the mole ratio of mono- and bifunctional (polyfunctional) monomers used in the polymerization reaction. Polymers with crosslinking up to 8% are gel-type. Higher crosslinking, greater than 8%, leads to the formation of macroreticular structures (a mixture of larger and very small pores) resulting in a high density of polymer chains. For example, poly(styrene-co-divinylbenzene) can have crosslinking up to 40% (commercial ion exchange resins, e.g., [118]) and can reach as high as 80% in some cases [119].

Unfunctionalized polymers are primarily used in the production of various polymer goods and construction/packaging materials. However, for applications in physical–chemical processes (adsorption and catalysis), the presence of functional groups in polymer is necessary. These functional groups can be introduced by two basic routes as follows:

- Functionalization of a pristine C-H type polymer.
- Production of a polymer by copolymerization of functional monomers (route **B** in Figure 3), i.e., monomers containing functional groups with heteroatoms, which exhibit a significantly higher polarity compared with moieties having only C and H atoms (e.g., -SO_3_H in comparison with C-C and C-H parts).

Given that monomers with functional groups are commonly more expensive than unfunctionalized monomers, the functionalization of a previously prepared polymer is a common approach (see Figure 3). Typical functional polystyrene materials, which are also suitable for the catalysis and then possible preparation of dispersed metal catalysts, include [120] the following:

- Strong cation exchangers (anionic polymers, R = SO_3_H) prepared by sulfonation (sulfuric acid or combination with SO_3_).
- Strong anion exchangers (R = [N(R^1^,R^2^,R^3^)]^+^A^−^, where A^-^ represents HO^−^, Cl^−^, or another anion) prepared by chloromethylation (e.g., chloromethyl methyl ether) and subsequent reaction with a tertiary amine.

**Figure 5:**
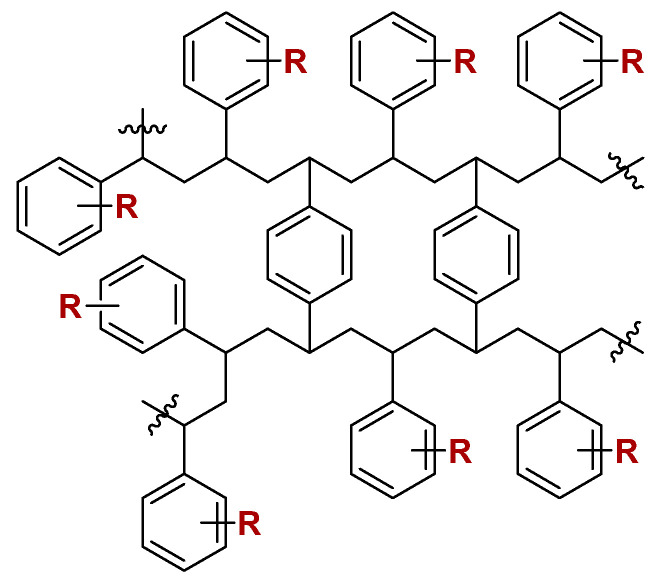
Functionalized poly(styrene-co-divinylbenzene), **R** represents -SO_3_H, -[N(R^1^,R^2^,R^3^)]^+^A^−^, or other functional groups.

The structures of sulfonated poly(styrene-co-divinylbenzene) (SPSDVB) and tetraalkylammonium poly(styrene-co-divinylbenzene) (APSDVB) are depicted in Figure 5.

From an application point of view, the effect of crosslinking on temperature resistance is significant. According to Li et al. [121], when crosslinking (mole content of divinylbenzene) in a porous polymer poly(styrene-co-divinylbenzene) was less than 10%, a melting process was observed as an endothermic event at about 270 °C. However, with crosslinking above 15%, no melting was registered, and an exothermic phenomenon occurred between 250 and 350 °C. It is also important to note that the presence of functional groups can decrease temperature stability. For example, sulfonated PSDVB has an application temperature limit of about 130 °C.

The typical polymerization of vinyl monomers involves various kinds of initiators to trigger the polymerization reaction. Among these, dibenzoyl peroxide (DBPO, C_6_H_5_-(CO)-O-O-(CO)-C_6_H_5_, giving a benzoyl radical) and azobisisobutyronitrile (AIBN, (CH_3_)_2_(CN)C-N=N-C(CN)(CH_3_)_2_), a providing *tert*-butyl nitrile radical) are the most frequently used for radical polymerization. Polymerization can be performed using the following methods:

- Block polymerization.
- Dispersion polymerization. This method involves the use of water–oil systems, where some surfactants (e.g., dodecylbenzene sulfonate) and proper mixing are necessary to obtain regular low-size dispersion particles [122].
- Other special types of polymerization. For monomers with different reactivities, such as those containing low polar groups (C-H vinyl compounds) and more polar groups (e.g., -SO_3_H), special procedures involving electromagnetic microwaves [123,124] or gamma irradiation [100,101,125,126] are applied.

Ion exchange resins are an important group of functionalized polymers. There are several commercial suppliers of ion exchange resins, e.g., the DuPont Amber series (formerly sold as DOWEX) [118], Resindion SRL (a subsidiary of Mitsubishi Chemical Corporation) [127], Purolite (an Ecolab Company) [128], and others. These ion exchange resins can act as acid or base catalysts, and they are also important for anchoring metal cations into the polymer network through the ion exchange process.

It is important to note that PSDVB is able to encapsulate nanoparticles (NPs). These NPs are solely stabilized by the steric factor or electrostatic interaction of benzene rings, resulting in excellent catalytic activity due to the absence of strong ligands on their surfaces [129].

There are also more complex functionalizations of vinyl polymers, e.g., surface oxidation of PE fibers (formation of carboxylic groups), conversion of carboxylic acid groups to acid chloride (using thionyl chloride), and, afterward, attachment of a branched polyester with free carboxyl groups. This material enables the easy adsorption (ion exchange) of palladium from Na_2_PdCl_4_ to prepare catalytically highly active nanoparticles [130].

However, post-functionalization often leads to an uneven distribution of functional groups, with a higher concentration generated near the surface. This issue can be addressed by using functional monomers during the polymerization process. Commercial monomers such as acrylic acid and its derivatives, mainly esters and nitrile, are produced on a large scale. These monomers are used in polymerization processes, yielding polyacrylic acid (PAA), poly(methyl acrylate) (PMA), poly(butyl acrylate) (PBA), and poly(acrylonitrile) (PAN). Vinyl acetate is particularly important, as it can be polymerized into poly(vinyl acetate) (PVAc), which, upon hydrolysis, provides poly(vinyl alcohol) (PVA). PVA is highly suitable for the stabilization of nanoparticles [131,132]. The application of polyacrylic acid (PAA) and its derivatives in combination with crosslinking agents, such as *N*,*N*’-methylenebisacrylamide, as supports for catalysis or in the formation of catalytic composites began much later compared with the earlier adoption of sulfonated poly(styrene-co-divinylbenzene) (SPSDVB) and tetraalkylammonium poly(styrene-co-divinylbenzene) (APSDVB) [8,110,133,134,135,136,137,138,139].

**Figure 6:**
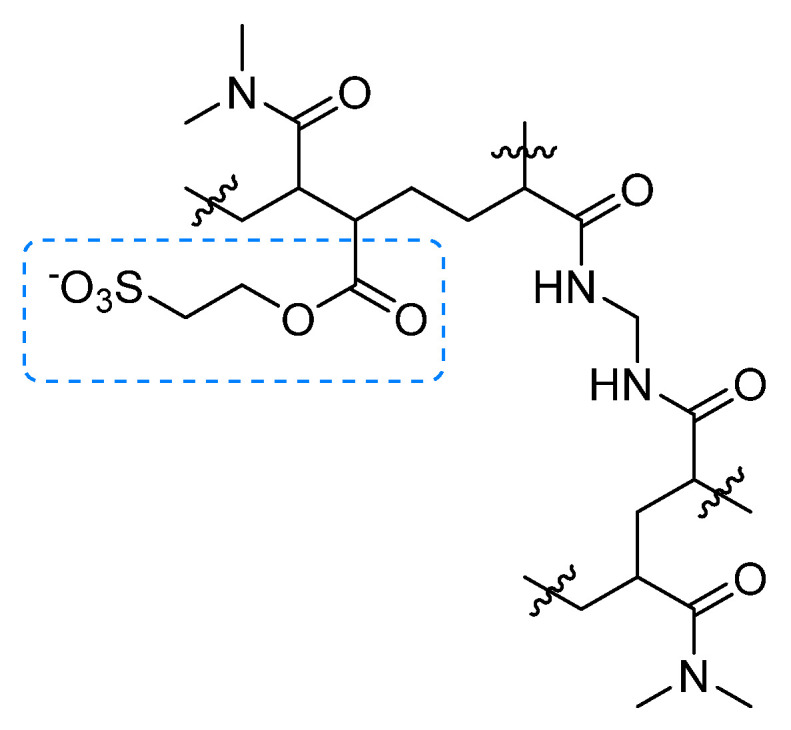
Segment of a microporous poly-(*N*,*N*-dimethylacrylamide)-(1-methacryloyl-ethylene-2-sulfonate)-(*N*,*N*’-methylenebisacrylamide) resin [101].

Acrylic polymers (mentioned above) are produced mainly through initiated polymerization processes. However, when there is a significant difference between the reactivity of monomers, particularly when their reactivities differ by several units or more, special polymerization techniques must be used in the production process. Figure 6 illustrates a segment of resin prepared from functionalized monomers using gamma irradiation, which achieved a very homogeneous distribution of functional groups. The importance of the -SO_3_^−^ functional group is particularly emphasized in this context.

**Figure 7:**
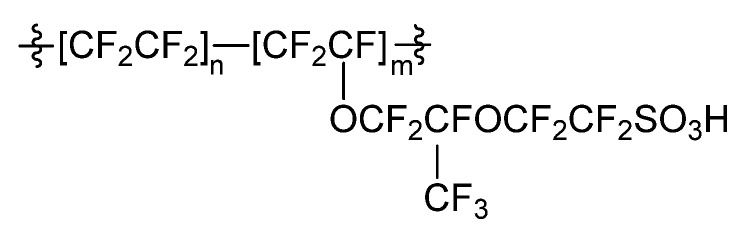
Nafion structure adopted from [144].

Rather stable functional polymers prepared from vinylpyridines and vinylpyrrolidones allow the formation of stable dispersed metal catalysts [91,140,141,142,143]. A peculiar class of functionalized vinyl polymers is prepared from perfluorinated vinyl monomers combined with other functional compounds. Figure 7 depicts the structure of classical Nafion-type polymer synthesized from tetrafluoroethylene, perfluorovinyl ether, and perfluoro(4-methyl-3,6,-dioxaoct-7-ene) sulfonic acid (CF_2_=CFOCF_2_CF(CF_3_)OCF_2_CF_2_SO_3_H) [144]. These types of polymers are very important for use as membranes in electrolytic processes and fuels cells [137,145].

### 3.2. Condensation Polymers

Condensation polymers are formed in a polycondensation process in which a low-molecular-weight byproduct, most often water, is generated. The most important groups of polycondensation products include the following:

- Polyesters;
- Polyamides;
- Formaldehyde resins;
- Polyanilines;
- Polybenzimidazoles;
- Porphyrins and phthalocyanines.

**Scheme 1:**
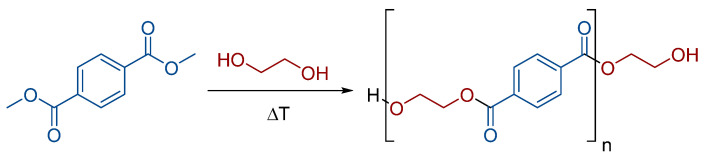
Formation of poly(ethylene terephthalate).

A typical example of polyester is poly(ethylene terephthalate) (PET), which is produced from terephthalic acid (TA) and ethylene glycol (EG, 1,2-ethanediol), as illustrated in Scheme 1. Alternatively, dimethyl terephthalate (DMT) can be used instead of pure TA, as the removal of methanol formed during the reaction is easier because of its lower boiling point (65 °C) compared with water (100 °C).

**Scheme 2:**
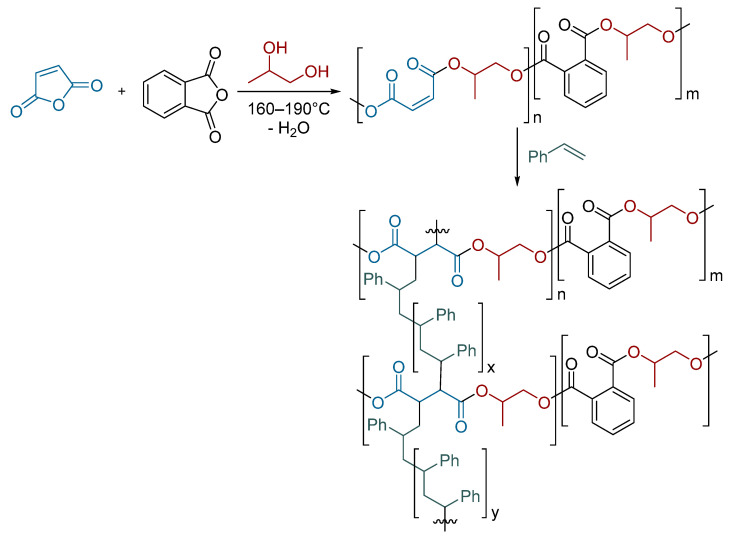
Crosslinked polyester resin adopted from [147].

The utilization of pure PET for catalysis purposes is relatively uncommon. More frequently, unsaturated polyesters, which allow for further crosslinking (Scheme 2), are mostly employed in the production of composite materials. However, modified PET has been successfully used in the catalytic hydrogenation of 4-nitrophenol [146].

**Figure 8:**
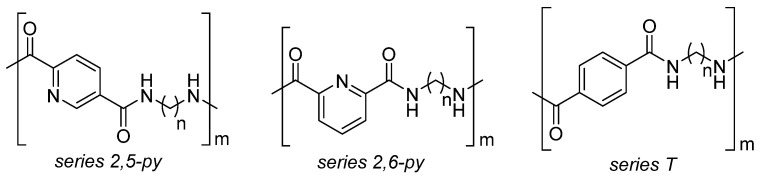
Segments of polyamide resins with increased functionality (in the case of pyridine substituents) adopted from [149].

Polyamides (PAs) are characterized by the presence of a more active functional group (-CO-NH-) compared with the ester group (-CO-O-) found in polyesters. A significant feature of polyamides, particularly aromatic polyamides, is their capability to form rather strong bonds among polymer chains through hydrogen bonding, which enhances a material’s mechanical properties (e.g., aramid fibers) [148]. PA can be prepared through several methods. One common approach is the ring-opening polymerization (ROP) of lactams, such as in the production of Nylon 6. Another method involves the condensation reaction between bifunctional carboxylic acids and bifunctional amines, where water as the condensation byproduct is produced, as seen in the production of PA 6,6; PA 6,8; and related polymers. Alternatively, PA can be prepared using certain chloroderivatives, such as in the production of aramid from 1,4-phenylenediamine (para-phenylenediamine) and terephthaloyl chloride (HCl is formed as the condensation byproduct). Figure 8 illustrates PA resins commonly used in the preparation of metal catalysts.

The strong affinity of metal particles to -NH-CO- groups of polyamides makes these materials excellent candidates for the preparation of metal-supported catalysts [149,150,151,152].

**Figure 9:**
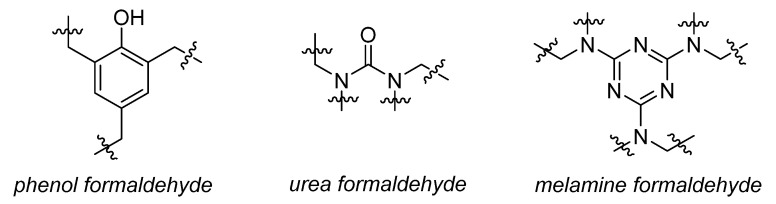
Formaldehyde-type resins adopted from https://www.britannica.com/science/aldehyde-condensation-polymer (accessed on 26 June 2024).

The generation of resins with functional groups through reaction with formaldehyde varies depending on the second reactant. For example, in phenol–formaldehyde resins (Figure 9), the functional group is -OH. However, this group has poor accessibility when the resin is thermally converted into Bakelite, the first synthetic plastic developed in 1907. 

Urea–formaldehyde and melamine–formaldehyde resins are more interesting for catalytic applications (Figure 9) [153,154,155,156,157]. Notably, even pure melamine can serve as a support for catalytic species [158].

**Figure 10:**
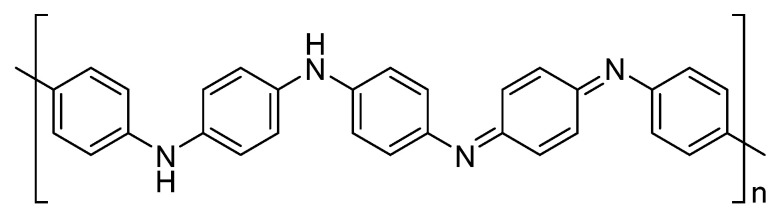
A simplified structure of polyaniline.

Polyanilines (PANIs, Figure 10) are a type of condensation polymer with notable properties and applications. There are two primary methods for the formation of polyanilines [159] as follows:

- Chemical oxidation;
- Electrochemical oxidation.

PANIs offer several advantages including poor solubility, rather good thermal stability (up to 200 °C), and electrical conductivity. These properties arise from PANIs’ strongly conjugated π electron system and the rigidity of their molecular backbone. Structured/porous PANI materials are used as adsorbents [160,161] and composite materials for the preparation of catalysts, including electrocatalysts [162,163,164,165,166,167,168]. 

**Scheme 3:**
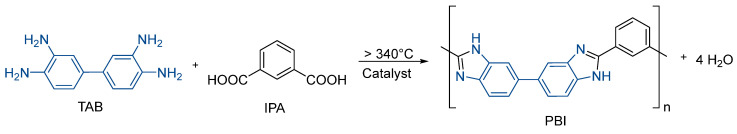
Synthesis of polybenzimidazole (PBI) starting from 3,4,3′,4′-tetraminobiphenyl (TAB) and isophthalic acid (IPA, 1,3-benzene dicarboxylic acid), adopted from [169].

The group of condensation polymers also includes polybenzimidazoles (PBIs, Scheme 3). 

Similar to the previous examples, carboxylic acid derivatives can also be used in the synthesis of PBIs (e.g., diphenyl ester of IPA), instead of pure dicarboxylic acid [169,170]. These polymers are much more thermally stable compared with other condensation polymers, and their -N-C=N- functional groups exhibit a good chelating capability for metal cations. These characteristics make PBIs suitable for application in fuel cells [171,172,173] as well as for catalysis [174,175,176,177]. The amphiphilic and other properties of PBIs are well documented in the literature [178].

### 3.3. Polyurethanes and Polyureas

**Scheme 4:**
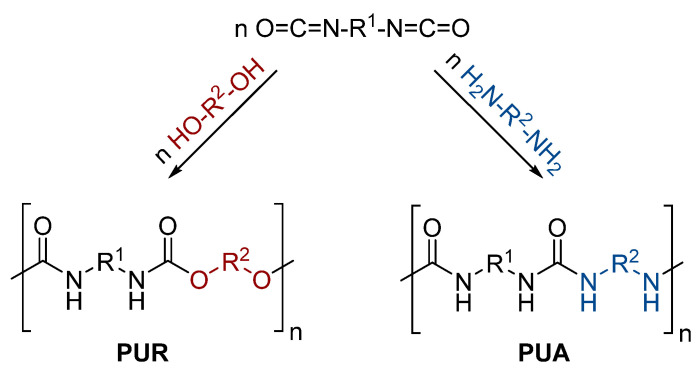
Formation of PUR and PUA.

Polyurethanes (PURs) and polyureas (PUAs) are types of addition polymers characterized by urethane and urea linkages, respectively. The synthesis of PURs and PUAs does not produce side products compared with the formation of other polymers. Unlike vinyl polymers, different reaction groups are required for the polymerization reaction, similar to polyesters and polyamides. A common (industrial) preparation of PURs and PUAs uses diisocyanates as starting materials. The reaction of diisocyanates with polyols and diamines yields PUR and PUA, respectively (Scheme 4). Further details about catalysts used in PUR synthesis [179] and the application of PUR-based supports in metal catalysis is described in the literature [180,181,182].

**Figure 11:**
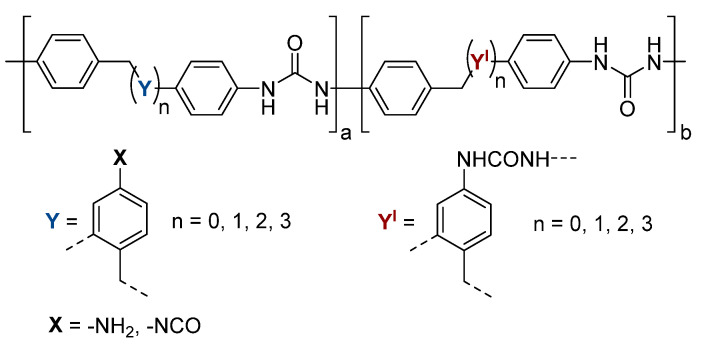
Structure of the matrix used for the commercially available EnCat catalyst [183].

**Figure 12:**
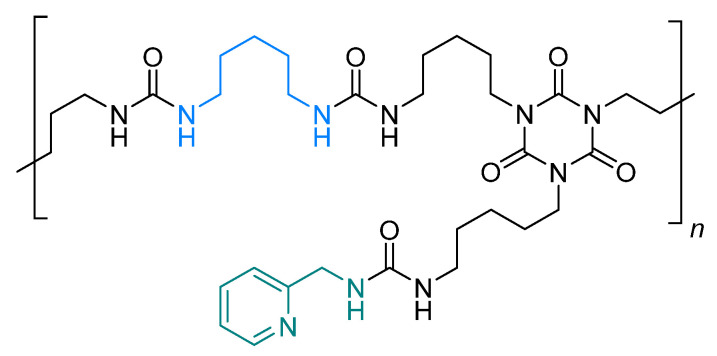
Structure of the PUA matrix prepared from PDI trimer (Desmodur Eco N 7300, Covestro AG) and cadaverine (1,5-diamine pentane) and modified with 2-(aminomethyl)pyridine [112].

Figure 11 and Figure 12 illustrate PUA matrixes used for supported metal (palladium) catalysts, which were used in aminocarbonylation reaction systems [112].

Because of the commercial availability of PUA catalysts [183], a broad range of applications has been reported [113,184,185,186,187,188,189].

### 3.4. Epoxide Polymers

**Figure 13:**
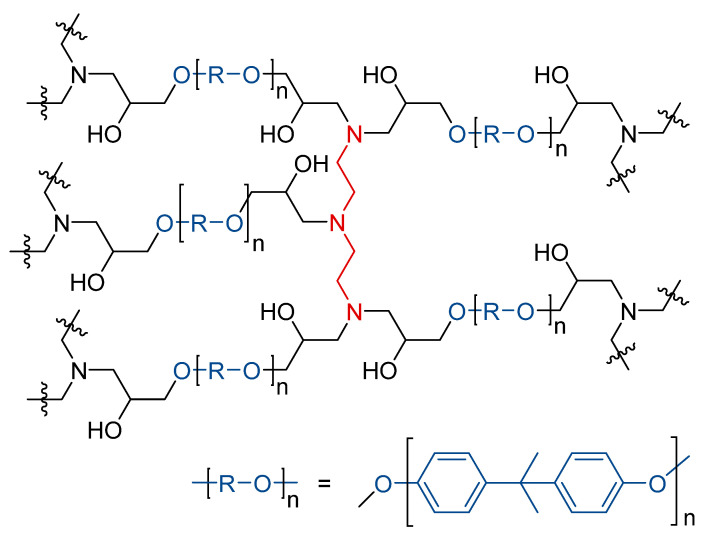
Structure of an epoxy resin with diethylenetriamine (red), R represents the Bisphenol A skeleton (blue, C_6_H_4_-C(CH_3_)_2_-C_6_H_4_).

Most epoxide polymers are prepared by the condensation of Bisphenol A (4,4′-(propane-2,2-diyl)diphenol) with epichlorohydrin (2-(chloromethyl)oxirane), followed by crosslinking using polyamines (Figure 13). These polymers exhibit specific properties such as rigidity and toughness, classifying them as thermosets. However, if a porous structure of resulting polymer is required, porogenic agents (non-reactive liquids) have to be used in the polymerization process [190]. Because of their high thermal stability, epoxide resins are suitable as supports (enzymes, dispersed metals) for catalysts [191,192,193,194,195,196].

### 3.5. Phosphine Polymers

**Scheme 5:**
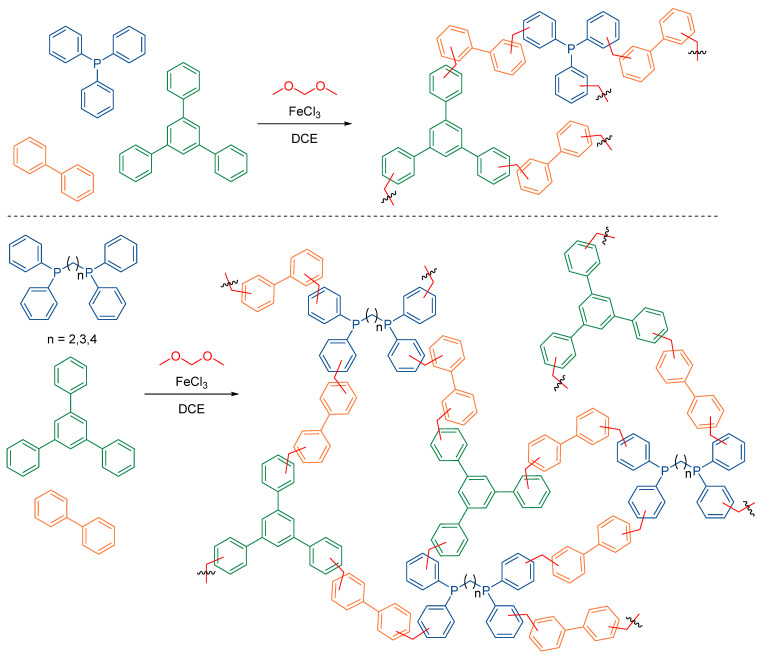
Synthesis of a phosphine polymer (adopted from [197]).

Phosphine ligands such as triphenylphosphine are well-known for their ability to stabilize homogeneous metal catalysts in various chemical processes. This property makes them ideal core structures for designing and preparing phosphine polymers as supports for metal catalysts. Scheme 5 illustrates the heterogenization of triphenylphosphine-based ligands including the crosslinking process [197]. Pd catalysts prepared using this support showed very good selectivity and stability, as well as recyclability in Suzuki–Miyaura reactions.

### 3.6. Natural Polymers

**Figure 14:**
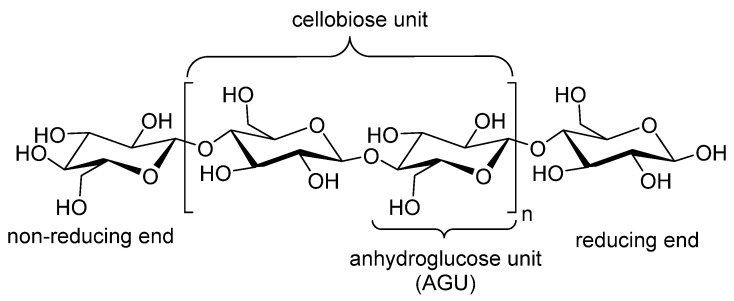
Cellulose polymer chain.

**Figure 15:**
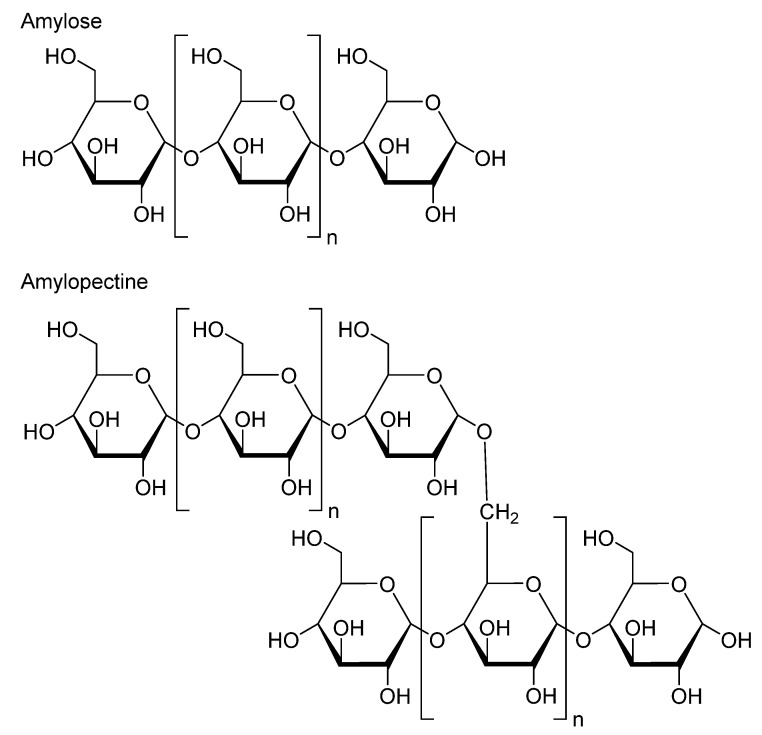
Components of starch: amylose and amylopectin.

**Figure 16:**
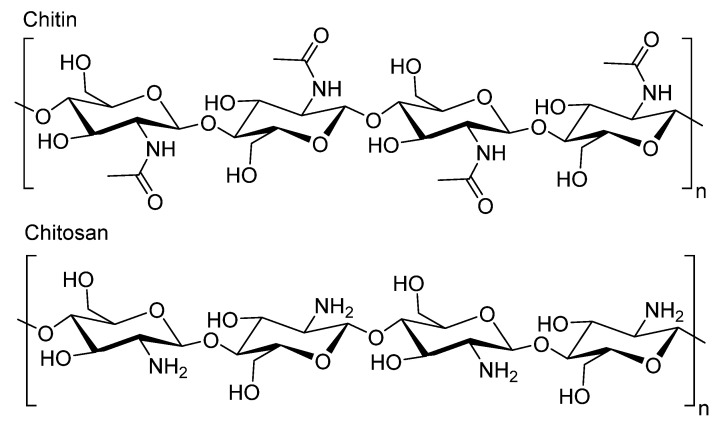
Structure of chitin (upper) and chitosan (down).

Natural polymers serve as viable alternatives to synthetic ones, as shown in Figure 3. The main representatives include cellulose (Figure 14), starch–pectin, amylose and amylopectin (Figure 15), and chitin and chitosan (Figure 16).

Cellulose is sourced from trees and plants, which also contain other organic (e.g., hemicelluloses, lignins) and inorganic components. To isolate cellulose, it is necessary to remove, or significantly decrease the content of these other organic materials. This process is typically achieved through pulp preparation for making cellulose papers [198] or by using other specialized technologies [199], providing specific composite materials if needed. Cotton, obtained from cotton plants, represents a form of virtually pure cellulose. Recently, there has been an increasing interest in the production of cellulose using microbial processes [200]. Cellulose forms stable crystalline structures because of the regular positions of -OH groups and hydrogen bonding, which makes it quite an efficient adsorbent and catalyst support, particularly when functionalized [71,72,201,202]. Recent trends focus on the functionalization of biomass, particularly wood-based materials, for use as supports in multifunctional catalysts [96,203,204,205]. Biochar, obtained from biomass through pyrolysis at elevated temperatures (up to 500 °C), has also attracted attention for its use in adsorption processes [206,207,208] and catalysis [209,210,211]. The treatment of biomass components at higher temperatures (>700 °C) enables the preparation of activated charcoal, suitable for adsorption and as a support for metal catalysts [212].

Starch, primarily obtained from potatoes and maize [213], is relatively soft compared with cellulose and readily undergoes hydrolysis. Consequently, it requires functionalization before its catalytic application [214,215].

Chitin and chitosan are usually isolated from marine animals (e.g., lobsters, crabs, shrimps), other invertebrates (e.g., scorpions, spiders, ants), and microorganisms (e.g., green algae, yeasts, fungal cell walls). The most established technology involves the isolation of chitin and chitosan from the waste products of marine animal processing [216,217]. The amino groups present in the chitin and chitosan structures enhance their functionalities compared with cellulose and starch. As a result, chitin and chitosan have found wider applications in catalysis, mainly as supports for metal complexes and dispersed metals [216,218,219,220,221,222,223,224,225,226]. 

A general disadvantage of biomass-derived catalytic components is their reproducibility (in production and application), which depends on the source, composition, and morphology, as well as the treatment protocol. Despite this challenge, their low cost drives increasing exploitation. 

Additionally, the direct use of CO_2_ for functional polymer preparation [227] also falls under the category of renewable polymers.

### 3.7. Conductive Polymers (CPs)

The polymers discussed earlier, except for polyaniline, are not electrically conductive. The following section focuses on OMOPs that exhibit (semi)conductivity, and a few key points on this topic are outlined below.

The electrical conductivity of a material depends on the energy gap between its valence band and conduction band, known as the band gap or forbidden energy gap (*E*_g_) [228]. A larger *E*_g_ (measured in eV) corresponds to lower electrical conductivity because more energy is required for electrons to transition from the valence band to the conduction band. In conductors, the upper energy levels of the valence band overlap with the lower levels of the conduction band, allowing electrons to move freely through the material. Based on electrical conductivity, materials are classified into three groups (where band energy gap—*E*_g_ (eV) and conductivity—σ, (S m^−1^) are measured at room temperature) as follows:

- Insulators: *E*_g_ > 3.6; σ < 10^−6^, e.g., PE and PP (10^−20^), PET (10^−21^), and Teflon (10^−24^).
- Semiconductors: 0.17(InSb) < *E*_g_ < 3.6 (ZnS); 10^−6^ < σ < 10^5^, e.g., germanium (2–1000), silicon (1.67 × 10^−2^ to 10), and organic polymer semiconductors (CPs, see below).
- Conductors: *E*_g_ = 0; σ > 10^5^, e.g., silver (6.30 × 10^7^) and copper (5.96 × 10^7^).

The electrical conductivity in semiconductors is more complex than in conductors [228,229]. Charge carriers consist of electrons in the conduction band and “holes” in the valence band, which are generated by external energy sources such as electricity (e.g., light-emitting diodes) or photons (e.g., photovoltaic cells). These charge carriers move in opposite directions, and electrons can eventually recombine with holes, releasing energy in the process.

CP polymers are categorized into two main types [230] as follows:

- Intrinsic (ICPs): electricity is conducted by electrons and holes, facilitated by the presence of conjugated π-bonds.
- Extrinsic (ECPs): dopants are added, making conjugated double bonds (redox doping), or non-redox doping, photo doping, and charge-injection doping.

A special group includes the following:

- Holes: created by removal of electrons during doping.

**Figure 17:**
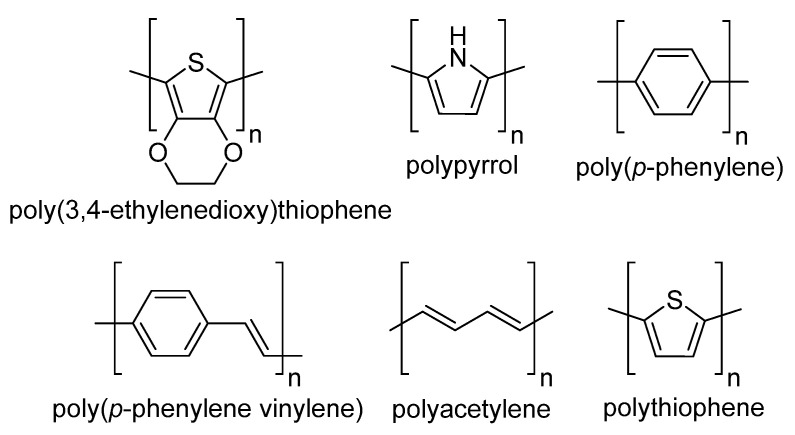
Examples of intrinsic conductive polymers.

One of the oldest intrinsic conductive polymers is polyaniline (PANI, Figure 10), which was first synthesized by Letheby in 1862 via the anodic oxidation of aniline in sulfuric acid [231]. Figure 17 shows other common ICPs. Most of these polymers, except for polyacetylene (produced by the polymerization of acetylene), can be synthesized through the oxidative polymerization of their corresponding monomers. This reaction is considered a “condensation” process, as it involves the release of hydrogen [232].

Extrinsic conductive polymers (ECPs) can be produced from nonconductive polymers by incorporating dopants, particularly carbon-based materials such as carbon black, graphite, carbon nanotubes, and graphene. Metal nanoparticles, including silver, copper, aluminum, and nickel, also enhance the conductivity of polymers. In large-scale applications like tire manufacturing, carbon black serves not only as a conductive agent (helping eliminate static electricity) but also improves the mechanical strength of the final product. For example, [233] describes the preparation and characterization of conductive polymers designed for electromagnetic (ELM) energy absorption. Nanoparticles, including quantum dots (e.g., hydrogenated BaTiO_3_), are incorporated to create high-quality ELM absorbent materials.

Non-conductive polymers (NCPs), such as vinyl polymers, are generally unsuitable for electronic, photoelectronic, electrochemical, and photochemical applications. Even intrinsic conductive polymers (ICPs) require atomic-level modifications to optimize their properties for specific uses. Various chemical agents, such as halogens (I_2_, Br_2_), hydrogen halides (HF, HI, HCl), alkali metals (Na, K), Lewis acids (FeCl_3_, NH_4_BF_4_), and others, are commonly employed for these modifications [231,234].

According to the classification of conductive polymers (with conductivity ranging from 10⁻⁶ to 10⁵ S m⁻¹), metal–organic polymers, covalent organic frameworks (COFs), and metal–organic frameworks (MOFs) can also be included in this category, as well as catalysts derived from them (covered in subsequent sections).

Conductive polymers are primarily applied in electronics, electronic devices, and energy storage. Their uses include the fabrication of touch screens and tactile sensors [235,236], specialized transistors and switches [237], chemical sensors [238], capacitors [230,239], and solar cells [240]. In electrochemical applications, significant attention has been focused on water splitting [241,242,243,244] and CO_2_ utilization, aiming to mitigate the greenhouse effect [245,246,247,248,249,250,251]. Photocatalytic processes for addressing CO_2_-related challenges are also being investigated [251,252,253,254,255]. Additionally, the advantages of conductive polymers have been demonstrated in medical and healthcare applications [256,257,258,259,260].

### 3.8. Covalent Organic Frameworks (COFs)

**Scheme 6:**
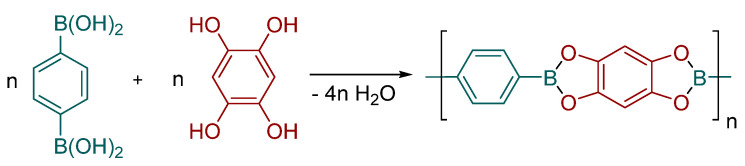
Formation of a linear (1D) COF.

COF materials can be synthesized using the principles of reticular chemistry, which involves linking molecular building blocks through strong bonds to create regular (crystalline) open frameworks [261]. This process, known as “self-assembling”, requires the synthesis to proceed over a specific time scale that allows thermodynamic processes to form such regular structure [262]. Scheme 6 illustrates the construction of a linear COF. 

**Figure 18:**
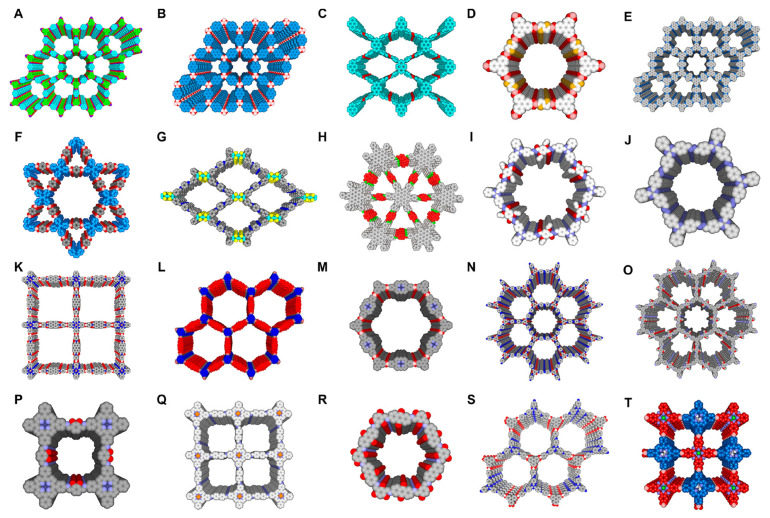
Examples of 2D COFs with layered stacking structures for (**A**) TP-COF, (**B**) PPy-COF, (**C**) Py-Azine COF, (**D**) T COF 4, (**E**) CS-COF, (**F**) TPE-Ph COF, (**G**) TTF-Ph, (**H**) HBC-COF, (**I**) TFPT-COF, (**J**) N3-COF, (**K**) DTP-ANDI-COF, (**L**) RA-COF, (**M**) TP-POR COF, (**N**) TATTA-TPBA COF, (**O**) TPB-DMTP-COF, (**P**) CuP-2,3-DHTP COF, (**Q**) COF-367-Co, (**R**) DAAQ-TFP COF, (**S**) HHTP-FPBA-TATTA COF, and (**T**) H2TPP-NiPc-COF (Reproduced from ref. [263] with permission of Royal Society of Chemistry.)

Two-dimensional (2D) and three-dimensional (3D) COFs are formed from building blocks bearing more functional groups. In addition to boron-containing COFs (Scheme 5), triazine, imine-based COFs, and others are also significant [261]. Figure 18 illustrates the structures of typical 2D COFs. The acronyms in this figure denote [249,263,264,265,266,267,268,269,270] the following: **TP**: Terephthalaldehyde; **PPy**: 1,4-benzenediboronic acid (BDBA) and pyrene-2,7-diboronic acid (PDBA); **Py-azine**: 1,3,6,8-tetrakis(4-formylphenyl)pyrene (TFPPy) and hydrazine; **T COF 4**: thioether-terminated and triazole-bridged; **CS COF**: prepared from C3-symmetric triphenylene hexamine (TPHA) and C2-symmetric *tert*-butylpyrenetetraone (PT); **TPE-Ph**: tetraphenylethene (TPE)-cored boronic acids (TPEBAs) and 1,2,4,5-tetrahydroxybenzene (THB); **TTF-Ph**: tetrathiafulvalene and THB; **HBC**: hexabenzocoronene and C2-symmetric benzene linker; **TFPT**: 1,3,5-tris(4-formyl-phenyl)triazine; **N3**: azine-linked Nx-COFs; **DTP-ANDI**: 2,3,6,7,10,11-hexahydoxytriphenylene and *N*,*N*’-di-(4-boronophenyl)-naphthalene- 1,4,5,8-tetracarboxylic acid diimide (NDIDA); **RA**: Rosmarinic acid; **TP-POR**: 2,3,6,7,10,11-hexahydroxytriphenylene and 2,7-pyrenediyldiboronic acid derivatives; **TATTA-TPBA**: 2,4,6-triphenyl-1,3,5-triazine triamino, 2,4,6-triphenyl-1,3,5-triazine trialdehyde, and 1,3,5-tris(4-carboxyphenyl)benzene derivatives, **TPB-DMTP**: triphenylbenzene and dimethoxyterephthaldehyde; **CuP-2,3-DHTP**: copper porphyrin and 2,3-dihydrooxyterephthalate; **COF-367-Co**: tetraaminophenyl porphyrin cobalt (II) and biphenyl_4,4′-dialdehyde; **DAAQ-TFP**: 2,6-diaminoanthraquinone and 2,4,6-trihydroxybenzene-1,3,5-tricarbaldehyde; **HHTP-FPBA-TATTA**: 2,3,6,7,10,11-hexahydroxytriphenylene and 4-formylphenylboronic acid and 2,4,6-triphenyl-1,3,5-triazine triamino; and **H2TPP-NiPc**: porphyrin-5,10,15,20-tetrayltetrakis(benzene-4,1-diyl))tetraboronic acid and octahydroxy nickel porphyrin.

The synthesis of COFs is typically a “bottom-up” process, where the COF structure is built from molecular building blocks. However, for specific applications, such as electrocatalysis, “top-down” techniques are employed [271].

COF materials typically feature a high specific surface (up to approximately 3000 m^2^ g^−1^), which results in a high adsorption capacity for gases (e.g., storage of hydrogen, methane, carbon dioxide, and ammonia). A reversible iodine capture utilizing amorphous-conjugated covalent triazine-based porous polymers is described in reference [272].

Semiconductive COFs containing certain photoelectric moieties may exhibit unique optical and electrical properties. Furthermore, their good thermal stability and resistance to hydrolysis [273] make COFs excellent targets for catalysis. This is particularly significant for nitrogen-containing COFs, such as those prepared from benzene-1,4 diamine and benzene-1,3,5-tricarbaldehyde. These COFs exhibit a high chelating capability for metal ions, allowing for the incorporation of metals such as palladium into the framework. For example, the Pd/COF-LZU1 catalyst has demonstrated excellent performance in coupling reactions [261].

Ionic COFs can be prepared by initial charging, post-modification, or deprotonation [274]. Conductive properties allow the materials to be utilized as ion conductors [275], sensors, tools for chemical analysis, etc. [274].

### 3.9. Metal–Organic Polymers

In this section, the following groups of metal–organic polymers are distinguished:

- Metal–organic monomers and polymers prepared by “classic polymerization” (distribution of metal moieties within the polymer is random).
- Metal–organic frameworks (MOFs), with a typical very regular morphology and topology and regular distribution of metal moieties.

#### 3.9.1. Polymers Prepared from Metal–Organic Monomers

**Figure 19:**
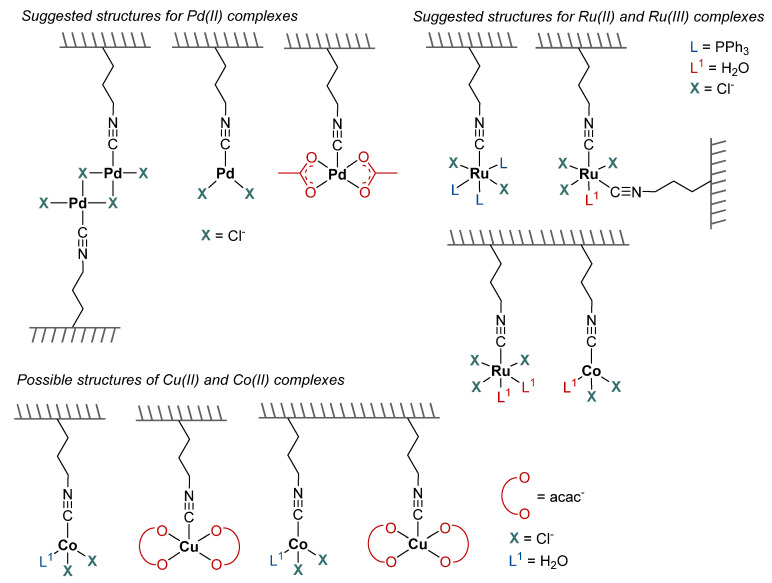
Isocyano metal complexes anchored to a polymer backbone (adopted from [83]).

The preparation of metal-containing functional polymers described in this section can be classified as route **D** (Figure 3). One of the earliest studies (1981–1984) on metal–organic polymers was devoted to the hydrogenation of olefins using Ru-immobilized complexes [276,277]. A comprehensive review of metal-containing monomers and their potential for polymerization is provided in the literature [278]. The development of metal–organic isocyanato complexes and their incorporation into a polymer backbone was described later [83,125,279,280] (Figure 19). 

**Figure 20:**
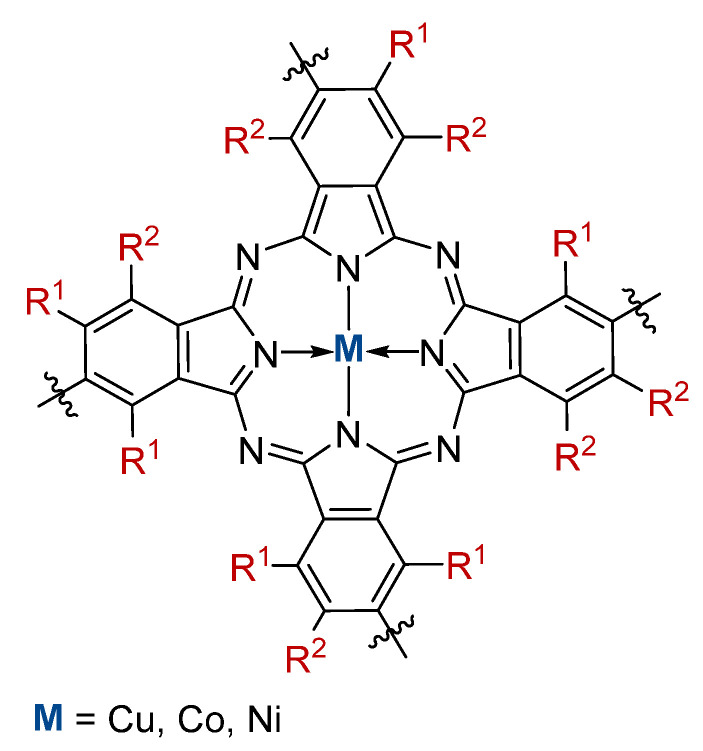
Structure of a polyphthalocyanine complex (adopted from [281]).

Another group of metal–organic monomers is represented by metal phthalocyanines [281]. Their monomeric compounds, including metal-containing variants, are significant biogenic species—such as the iron complex of a porphyrin derivative found in heme, the oxygen-carrying component of hemoglobin [282]. A new member of the porphyrin family was recently introduced [283]. These compounds have demonstrated excellent performance as oxidation catalysts under mild reaction conditions (up to 60 °C) [246,281,284,285,286,287,288] (Figure 20). 

Preparation of heterogeneous phosphinated polystyrene-bound Pd(II) complexes prepared by the reaction of phosphinated polystyrene with PdCl_2_ and aminated polystyrene-bound Rh_6_ and Rh_14_ carbonyl cluster complexes using Rh_6_(CO)_16_ as starting material are described in the literature [289]. The polymerizations of more sophisticated complexes, e.g., rhodium complexes bearing isopropenylphenyl diphosphines, are reported in [290]. For recent updates on inorganic and organometallic polymers, refer to [291,292].

#### 3.9.2. Metal–Organic Frameworks (MOFs)

MOFs are crystalline materials consisting of metal ions or clusters connected by organic linkers to form regular one-, two-, or three-dimensional structures. Because of their relatively simple synthetic preparation and diverse applications [86,258,293,294,295,296,297,298,299,300,301,302,303,304], MOFs have become a highly attractive research area over the past 40 years. A Web of Science search conducted on 23 July 2024 provided nearly 125 000 topics relevant to these materials. According to the website of novoMOF AG [305], more than 100,000 different MOFs had been synthesized by July 2024. Because of their similarity to zeolites [306], MOFs are considered a category of porous materials by the International Zeolite Association [307]. The vast amount of published data related to MOFs has prompted the use of artificial intelligence (AI) to facilitate simpler data mining and guide future research [308,309].

**Figure 21:**
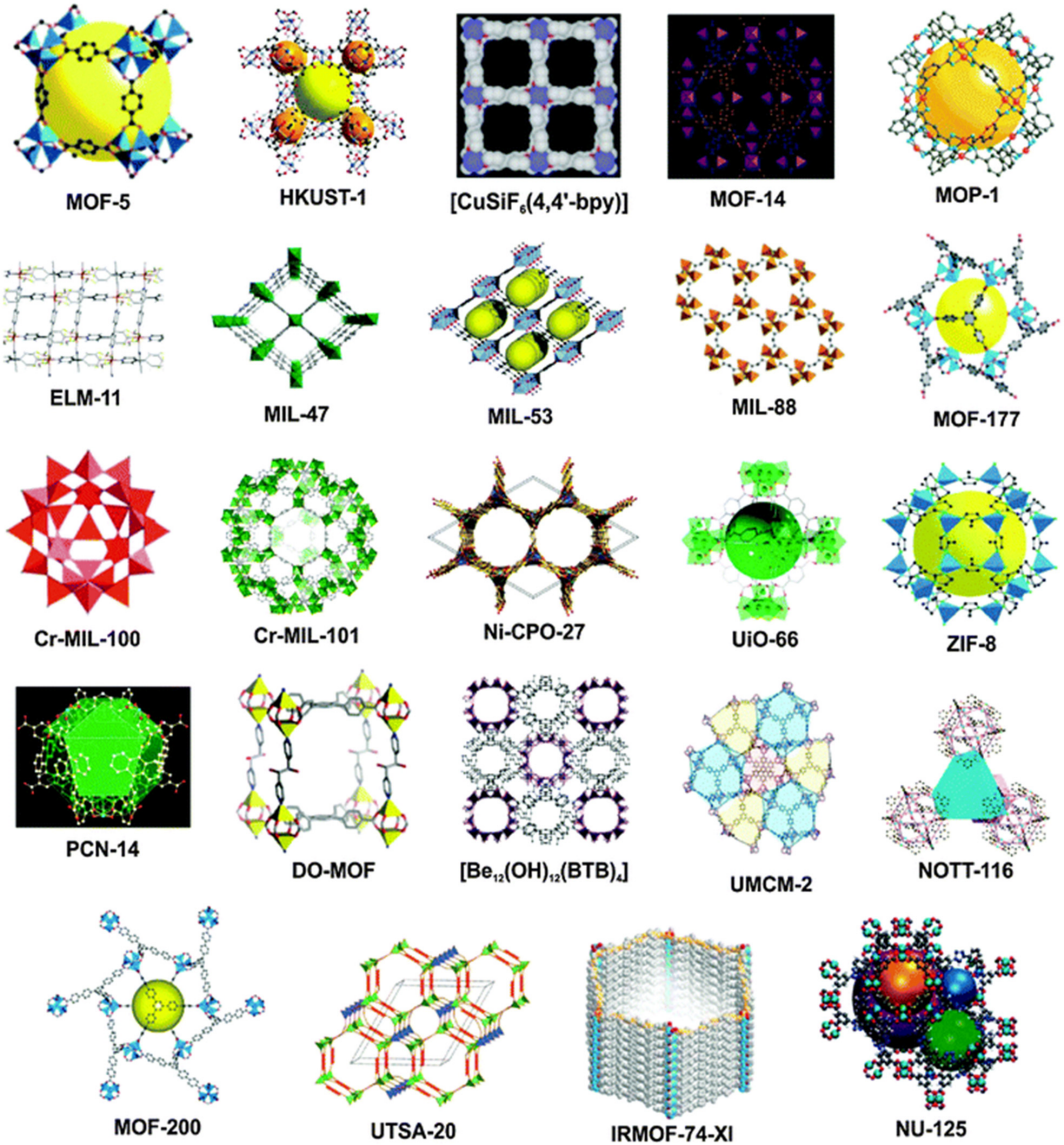
Porous MOFs prepared by several research groups with the aim of accommodating/retaining chemical species within their pores/channels. (Reproduced from ref. [327] with permission of the Royal Society of Chemistry.)

MOFs are usually synthesized using a metal salt/complex (all metals) and polyfunctional linkers, such as terephthalic acid (HOOC-C_6_H_4_-COOH, H2BDC), 1,3,5-benzenetricarboxylic acid ((HOOC)_3_C_6_H_3_, H3BTC) or other more sophisticated compounds following various methods (e.g., [310,311]). The synthetic preparation process, represented as route **F** in Figure 3, is guided by the principles of reticular chemistry [294,312,313,314,315], where reaction components are organized into a regular structure to achieve high crystallinity of a resulting material. Strategies for rational MOF synthesis, particularly those aimed at creating stable structures, often align with Pearson’s hard/soft acid/base (HSAB) principle, as discussed in the literature [316]. The final texture and properties of MOFs synthesized in liquid phases are significantly affected by the choice of solvent, mainly because of the solvent’s polarity, molecular size, and affinity of functional groups to the linkers [317,318,319,320,321]. Although defects in the crystal growth of MOFs [322] typically limit particle size to the micron and submicron ranges, these defects can also serve as sites for incorporating other additional components on the surface or within the cages of MOFs and COFs [34,323,324,325,326]. Figure 21 provides an overview of the basic structural segments of various MOFs [327]. 

The composition of MOFs in Figure 21 is as follows [328,329]: **MOF-5**, also known as **IRMOF-1**: Zn_4_O(BDC)_3_; **HKUST-1**: paddlewheel units of Cu_2_(COO^−^) surrounded by benzene tricarboxylate (BTC) ligands, Cu_3_(BTC)_2_; **MOF-14**: Cu(1,3,5-tris(4-carboxyphenyl)benzene); **MOP-1**: Cu_24_(m-BDC)_24_(DMF)_14_(H_2_O)_10_; **ELM-11**: Cu(BF_4_)_2_(4,4-bipyridine)_2_; **MIL-47**: [VO(BDC)](H2BDC)_5/7_; **MIL-53**: [Al(OH)(BDC)](H2BDC)_11/16_; **MIL-88**: Fe_3_(μ3-O)(BDC)_3_X(H_2_O)_2_, X = OH^-^/Cl^−^/F^−^; **MOF-177**: Zn_4_O(BTB)_2_; **Cr MIL-100**: Cr_3_OF(H_2_O)_2_ (BTC)_2_·nH_2_O (n = 28); **Cr MIL-101**: Cr_3_OX(BDC)_3_(H_2_O)_2_, X = OH^−^/F^−^; **Ni CPO-27**: CPO-27-M; M = Mg, Mn, Fe, Co, Ni, Zn; p-dobdc^4−^ = 2,5-dioxido-1,4-benzenedicarboxylate); **UiO-66**: Zr_6_O_4_(OH)_4_(BDC)_6_; **ZIF-8**: Zn(MIM)_2_, HMIM = 2-methylimidazole; **PCN-14**: H_2_[Co_4_O(TATB)_8/3_], TATB = (4,4′,4″-s-triazine-2,4,6-triyltribenzoate); **DO-MOF**: Zn_2_(TCBP)(DPED), H_4_TCBP = 1,2,4,5-Tetrakis(4-carboxyphenyl)benzene, DPED = 1,2-dipyridin-4-ylethane-1,2-diol; **Be_12_(OH)_12_(BTB)_4_**: H3BTB = 1,3,5-tris(4-carboxyphenyl)benzene; **UMCM-2**: Zn_4_O(T^2^DC)(BTB)_4/3_, H_2_T^2^DC = thieno [3,2-b]thiophene-2,5-dicarboxylic acid, H3BTB = 1,3,5-tris(4-carboxyphenyl)benzene; **NOTT-116**: Cu_2_(NTEI), NTEI^6−^ = 5,5′,5″-(4,4′,4″-nitrilotris(benzene-4,1-diyl)tris(ethyne-2,1-diyl))triisophthalate; **MOF-200**: Zn_4_O(BBC)_2_, BBC = 4,4′,4″-(benzene-1,3,5-triyltris (benzene-4,1-diyl))tribenzoate; **UTSA-20**: Cu_3_(BHB), BHB^6-^ = 3,3′,3″,5,5′,5″-benzene-1,3,5-triyl-hexabenzoate; and **IRMOF-74 XI**: (Mg/Zn)(linker), for more details see [330].

The shape and size of cages in MOFs depend on the type and length of linkers used, as nicely demonstrated by the IRMOF series (IRMOF-74-I to XI) [330]. For example, with the shortest linker, 2,5-dihydroxyphthalic acid (IRMOF-74-I), the typical cage size is around 1 nm. In contrast, with the longest linker, which consists of 11 substituted, linearly linked benzene rings with carboxylic acids at the first and last benzene rings (IRMOF-74-XI), the cage size expands to about 8 nm, containing 282 atoms in the ring. Another method for controlling the spatial distribution within MOFs involves different mixings of MOFs [331]. The accessibility of the interior space of a MOF strongly depends on the affinity the between reaction environment and the functional groups on the linkers. For instance, the effect of substituted organic linkers on the swelling (and consequent specific area) of MIL-88 in polar liquids (alcohols and pyridine) has been studied using *ex situ* synchrotron X-ray powder diffraction tests [332].

The majority of synthetic procedures for metal–organic frameworks (MOFs) follow a “bottom-up” approach, where organometallic polymeric frameworks are formed from molecular or ionic building blocks. However, a “top-down” approach, such as the preparation of nanosheets from bulk MOFs via delamination, has also been reported [333]. The key differences between these two methods are discussed in detail in references [311,334]. “Top-down” techniques commonly used for MOF fabrication include lithography, UV-lithography, nanoimprinting lithography, electron beam lithography, chemical etching, and UV/Vis–lithography [334]. In addition, “down-sizing” via milling is another method employed to create smaller MOF particles [335]. Generally, “bottom-up” methods are considered simpler and more versatile for the synthesis of MOFs, while “top-down” approaches are applied for specialized purposes, such as in electrocatalysis, sensors, adsorption, and other catalytic applications.

The high specific surface (up to 7000 m^2^ g^−1^) of MOFs and the possibility to tune the aperture size of their cages have predetermined them as good and selective adsorbents [261,297,298,327,336,337,338,339,340,341,342,343,344]. For potential industrial applications, ZIF-8 membranes with high specific area (up to 2400 cm^2^g^−1^) and well-tuned apertures have shown exceptional permeation selectivity for CO_2_/N_2_, with a ratio as high as 10,000, and the possibility to prepare spiral-wound membrane modules is noteworthy [320].

In terms of catalytic potential, the basic features of MOFs are similar to those of monomeric metal salts, such as zirconium carboxylates [345], which are relatively sensitive to temperature and species present in a reaction environment (e.g., water, acids, bases, and redox agents). Therefore, catalytic applications of simple-type MOFs are often limited because of the sensitivity of the metal–linker bond (e.g., metal–oxygen bonds in the case of polycarboxylic linkers). Simple carboxylates [345], for example, start to decompose around 100 °C, and copper-based MOF HKUST-1 decomposes at a temperature of 27 °C and 70% relative humidity after 50 days [346]. However, more complex MOFs have decomposition temperatures ranging from 300 to 500 °C [347], and imidazolate MOFs (ZIFs) are much more stable, surviving up to 500 °C in a N_2_ atmosphere [348]. Nonetheless, the decomposition temperatures of MOFs cannot be taken as the upper limit for their applications because chemical bonds can be broken by reactive species in the reaction environment, especially polar substances, such as water [349]. Available data from the literature [350] may be useful for the choice of MOF to be applied under certain conditions. A strong affinity of water to MOFs can be utilized for drying applications. For instance, MOF-303 has demonstrated a distribution coefficient for water in the MOF, compared with the water content in air, which exceeds 50. This finding has been validated through computer simulations and experimental data [351]. Additionally, a detailed description of water cluster formation in aluminum-based MOFs has also been discussed [352].

Similar to their temperature stability, ZIFs have demonstrated exceptional chemical stability, as evidenced by tests conducted in boiling benzene (7 days), methanol (7 days), and a sodium hydroxide solution (24 h). Crystallographic analysis revealed minimal structural changes following these tests [348]. The catalytic potential of ZIFs can be enhanced through the incorporation of various species, including functional groups, metal ions, and metal particles [353,354,355,356,357] as well as enzymes or enzyme mimetics [358,359,360,361]. Multicomponent Isoreticular MOFs (MIMOFs) represent a unique class of metal–organic materials, where multiple organic linkers and metal centers or clusters are chemically incorporated into a single molecular framework [362]. Such modifications allow for the customization of chemical properties, functional group modifications, and pore sizes while maintaining the structural integrity of the topology. As a result, materials with broader applications can be obtained. When zero-valent metal species are present on the cage walls of MOFs, the strong metal support interaction can occur [353,354,363], similar to those observed in catalysts supported on inorganic materials [364,365,366,367]. The chemical reactivity of ZIF-8 and MOF-5 has been successfully exploited in the sulfur vulcanization of styrene-butadiene rubber (SBR), offering a viable alternative to zinc oxide in conventional (CV) or effective (EF) curing systems [368].

MOFs have shown promising applications in photocatalysis and electrocatalysis, with processes operating at temperatures below 100 °C [243,244,369,370,371,372,373,374,375,376,377,378,379,380]. They also hold potential for use in fuel cells and batteries [381,382,383]. However, the effectiveness of MOFs in ”classical catalysis”, i.e., catalysis without additional magnetic or electromagnetic influence, remains a topic of ongoing debate [384,385,386,387,388,389,390].

MOF stabilization methods fall into the following categories: (i) “de novo” synthesis, which includes approaches such as forming multi-metal MOFs, incorporating hydrophobic ligands to enhance resistance to water attack, inserting stabilizing pillars, and creating interpenetrated frameworks, and (ii) post-treatment techniques, such as post-synthetic exchange (PSE), counterion replacement in ionic MOFs, and hydrophobic surface treatments [387]. Usually, these modifications hinder the movement of reaction species inside MOFs cages, thus decreasing the rate of a catalytic process. Unfortunately, quantitative data on these effects are scarce in the current literature. The increase in catalytic potential was also achieved by neutralizing acidic species within an MOF using ammonia [391]. A peculiar position belongs to MOF composites, e.g., with PBI, which increased proton conductivity as well as improved temperature stability (evaluated through DTA [392]). From a practical and economic standpoint, it is important to note that covalent organic frameworks (COFs) and metal–organic frameworks (MOFs) are generally more expensive than functional vinyl polymers or certain condensation and addition polymers, such as polyesters, amides, urethanes, and ureas. However, the potential use of COFs and MOFs for synthesizing more valuable specialty chemicals—such as jasminaldehyde and products from coupling reactions—expands their application potential [393,394].

Similarly, ionic MOFs can also be synthesized [395]. These can be categorized into cationic MOFs, where free-moving counter anions reside within the pores, and anionic MOFs, which contain counter cations in their pores. Through ion exchange, various functionalities can be achieved, making them suitable for applications such as adsorption, pollutant capture, storage, drug delivery, sensing, ion conduction, and heterogeneous catalysis [395,396]. Reference [396] summarizes the following results from catalytic experiments involving ionic MOFs (yields are in brackets): anionic rho-ZMOF, cyclohexane oxidation: 91%; anionic ZJU-28, hydrogenation of 1-octene: about 5000 TON; anionic bio-MOF-1, styrene epoxidation: 64–72%; cationic SLUG-21 and SLUG-21, ketalization of butanone: 71–97%; and cationic [Cu_2_L_2_(MeOH)_2_]_4_NO_3_H_2_O, Suzuki–Miyaura coupling: 43–98%. Cationic MOFs were successfully applied for the separation of biomolecules [397].

Despite the stability challenges and price, MOFs remain among the most extensively studied catalytic materials in recent years [87,353,354,356,360,396,398,399,400,401,402,403,404,405,406,407,408,409,410,411,412,413,414,415,416,417,418,419].

### 3.10. Generation of Metal Particles inside the Polymer Framework

Catalytic processes involving metal particles predominantly occur on clusters and surface defects such as kinks, edges, steps, and add atoms [420]. The specific concentration of catalytic sites (expressed per unit mass or unit volume of metal) increases as the particle size decreases. Moreover, the size and shape of (nano)particles significantly influence selectivity in a certain reaction [421,422]. Metal particles of nanometric dimensions are sometimes considered as a bridge between typical heterogeneous and homogeneous catalysis [423,424]. However, small-sized metal particles with a high specific surface tend to sinter and/or agglomerate because of the driving force to minimize system energy. Therefore, inorganic and organic supports with ligand groups that have an affinity for metal particles are used to prevent sintering and agglomeration [425,426,427,428]. Pore walls within carbon supports, zeolites, and MOFs as well as thick polymer networks can impede the growth and crystallization of metal particles, thus promoting the formation of smaller particles [45,109,134,135,429,430]. A particular challenge that remains is the stabilization of single atoms in single-atom catalysis [431,432].

As depicted in Figure 3, the generation of metal particles can proceed via four basic routes as follows:

- **A**—Charging a support with metal compounds (adsorption, ion exchange, coordinate species).
- **D**—Preparation of organometallic polymers.
- **F**—Generation of MOFs.
- **E**—Polymerization/stabilization of generated sub(nano) particles.

When metal precursors are embedded in a polymer matrix, they must be activated, typically through a reduction process. The reduction is usually carried out in a liquid phase using a compatible solvent that can penetrate the polymer’s interior. The reduction of metal cation depends on standard redox potentials of the metals and reductant [433,434]. The most suitable reducing agents are as follows:

- Borohydrides, typically NaBH_4_ in alcohol or water [100,101].
- Hydrazine hydrate [435].
- Hydrogen (in alcohols or water) [436].
- Alcohols, e.g., butanol [437].
- Aldehydes, particularly formaldehyde [438].
- Organic acids and sugars [438].

Metal particle size and the distribution of particles depend on the following:

- The “density” of a polymer network, implying resistance against the movement of metal precursors (cations, e.g., Pd[(L)_n_]^2+^, or anions, e.g., Pd[L)_n_]^(2−n)+^) and the reductant agent, e.g., BH_4_^−^.
- The concentration of metal precursors inside a polymer network and diffusivity in the swollen polymer.
- The concentration of the reductant and diffusivity in the swollen polymer.
- Polymer/catalyst particle size.

**Figure 22:**
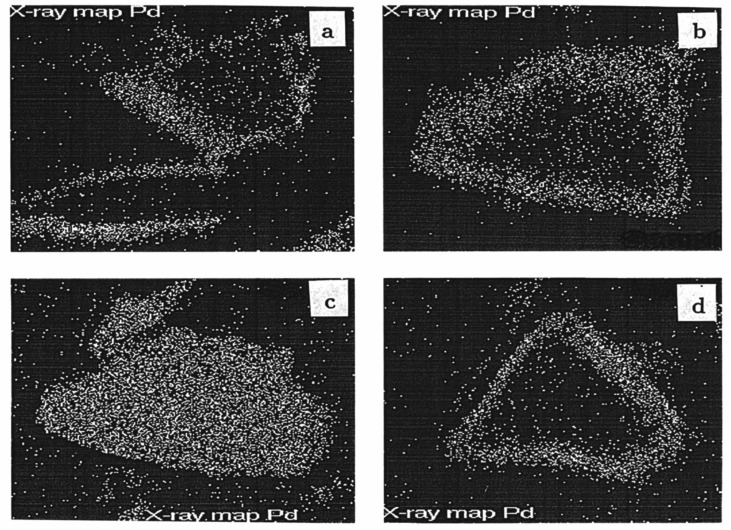
Distribution of palladium in microporous poly-*N*,*N*-dimethylacrylamide-*p*-styrylsulfonate-methylenebisacrylamide: (**a**) hydrogen in MeOH (100 kPa), (**b**) NaBH_4_ 0.066 M in water (10 times excess), (**c**) NaBH_4_ 0.0166 M in EtOH (10 times excess), and (**d**) NaBH_4_ 0.0189 M in EtOH (Reproduced from [101] with permission of Elsevier.)

When the concentration of the reductant is high, metal particles are generated close to the location of metal precursors inside the support, and the size of metal particles is governed by the elasticity of polymer chains (“willingness” to form a cage for the growth of a metal crystallite/agglomerate). As demonstrated in [100], palladium crystallites approximately 4 nm in size were generated within a dimethylacrylamide sulfonate resin through reduction with tenfold excess of NaBH_4_ relative to Pd^2^⁺. This process occurred independently of the metal content, which ranged from 0.5 to 8 wt.% of Pd in the final catalyst. However, a lower concentration of a reductant allows unreduced metal species to travel from the interior of a catalyst particle to its surface, as depicted in Figure 22. The uneven distribution of metal palladium was also observed in our recent research dealing with palladium catalysts supported on polyurea when a mixture of triethylamine, morpholine in 1,4-dioxane was used as a reducing agent [112].

In the case of MOFs, space limitations for metal crystallite growth are even more pronounced; e.g., TEM images of 1.0 wt.% Pd^0^ in UiO-67 revealed that the size of Pd particles was about 3 nm [439]. When metal particles were generated (through the reduction of a palladium dibenzylideneacetone complex with *n*-butanol) before the formation of a crosslinked polymer (poly(styrene-co-divinylbenzene) and embedded into a polymer network during polymerization, the size of palladium particles was significantly larger, about 50 nm [437].

Particle size can be also affected by ultrasonic waves. For example, copper particles prepared from copper acetate by reduction with citric acid in ethylene glycol decreased in size with increasing irradiation intensity [440]. However, it is necessary to note that this observation was for particles ranging from 167 to 520 nm, which are significantly larger compared with those generated within functional polymers.

### 3.11. Functional Groups

Non-functional organometallic polymers (OMOPs) are rarely used, as most contain heteroatoms that impart various functionalities. The functional groups in OMOPs can be categorized as follows:

- Directly catalytically active groups:
  ◦ Acidic groups (most frequently -SO_3_^+^H^+^ [441,442,443] and -OCF_2_CF_2_-SO_3_^−^H^+^ [444]);
  ◦ Basic groups (mainly -N(R^1^,R^2^,R^3^)^+^OH^−^ [445]) and nitrogen groups in organocatalysis, e.g., triazine [446], sulfur, phosphorous, and others [447];
  ◦ Bound metal–oxygen compounds (e.g., -WO_3_) and metal complex species (e.g., phosphine [197,448,449]).

Groups affecting the properties of metal nanoparticles, or their oxides, known as the metal–support interaction (MSI). This phenomenon is analogous to interactions seen with inorganic supports [366,450,451]. Depending on the nature of the interaction, MSI can be classified as strong metal–support interactions (SMSIs), medium metal–support interactions (MMSIs), and weak metal–support interactions (WMSI) [451]. In the context of OMOPs, covalent metal–support interactions (CMSI) are typical, facilitated by heteroatoms that can donate electrons from their lone pairs—most commonly nitrogen (e.g., -NH_2_, -NH—CO- in polyamides, polyurethanes, polyureas, and porphyrins) and phosphorus, as seen in MOFs [452,453]. Additionally, strong covalent interactions are prevalent in single-atom catalysts deposited on conjugated polymers [454]. For further details on functional polymers, refer to [455].

## 4. Characterization of Catalysts

The characterization of organic polymer catalysts is similar to that of inorganic catalysts; however, the stronger interactions between the reaction environment and the polymer network, compared with inorganic materials, must be considered. As a result, certain features—particularly those related to the accessibility of the interior space—cannot be accurately assessed when the supports or catalysts are in a dry state. For example, properties such as microporosity and the chemisorption of probes on the surface of metal particles (e.g., CO, H_2_ for estimating the specific surface area of dispersed metal) are affected. The following methods are used:

◦ *Texture and the Accessibility of interior space:*
  - Average size and distribution of carrier and catalyst particles (not metallic crystallites).
  - Optical microscopy.
  - Scanning electron microscopy (usually performed in conjunction with EDX).
  - Adsorption and desorption nitrogen, or krypton, isotherms to determine porosity, external and internal specific surfaces, and pore size distribution. The procedure is only suitable if the surface is accessible in the dry state of the catalyst (not suitable for gel-like polymer carriers).
  - Mercury porosimetry (only for mechanically stable solid materials and accessible pores).
◦ *Polymer catalysts or polymer carriers:*
  - Swelling.
  - SEC (Size Exclusion Chromatography) in the liquid phase [456].
  - ISEC (Inverse Steric Exclusion Chromatography) [457,458].
  - Diffusivity, measurement of transversal diffusion coefficient [459], or rotational mobility [58,460].

To evaluate the accessibility of the interior space of a swollen polymer, measuring swelling behavior is the simplest and most cost-effective method [54,100,101,125,140,461,462]. The solvent chosen for a reaction should ensure good accessibility without strongly attacking the polymer network.

In Professor Corain’s laboratory, special attention was given (1990–2014) to the swellability and accessibility of the interior space of OMOPs. Relationships between the concentration of polymer chains, transversal diffusion coefficient, and rotational behaviors of a probe molecule (TEMPONE) were found using Electron Spin Resonance (ESR) and Static Gradient Spin Echo Nuclear Magnetic Resonance (SGSE-NMR) [54,58,460,463,464,465,466,467].

The following group of characterization methods focuses on the chemical features of a polymer support and a prepared catalyst [100,104,108,151,189,194,226,279,297,314,328,468,469,470,471,472,473]:

- Chemical analysis of the carrier and catalyst with the deposited metal (most often decomposition in acid and then atomic absorption (AAS) or emission spectrophotometry–Inductively Coupled Plasma Optical Emission Spectroscopy and Mass Spectroscopy (ICP OES, ICP MS).
- X-ray reflection spectroscopy (XRF) to characterize surface composition.
- FTIR to characterize functional groups.
- Nuclear Magnetic Resonance (NMR) in a solvent and a solid state [474] to determine the structure of the carrier, including functional groups.
- Titration with basic components, e.g., NaOH solution to determine acidity.
- Adsorption measurements of basic components (e.g., NH_3_, organic amines) to determine acidity.
- Titration with acidic components, e.g., HCl to determine alkalinity.
- Adsorption measurements of acidic components, e.g., CO_2_ to determine alkalinity.
- X-ray powder diffraction (XRPD) to determine crystallographic phases and average particle size (Scherrer Eq.).
- Electron Diffraction X-ray Analysis (EDX) to determine components, including the metal distribution.
- Wavelength-dispersive X-ray spectroscopy (WDS, WDX), which is more sensitive than EDX.
- Transmission electron microscopy, including high resolution (resolution 0.1 nm) and scanning mode (TEM, HR TEM, STEM) to determine the particle size distribution of metal crystallites and arrangements of atoms/crystallographic phases.
- Scanning Tunneling Microscopy (STM) (resolution 0.1 nm with a 0.01 nm depth resolution); however, the surface has to be at least partially conducive. Suitable for observing details of MOFs and their composite.
- Atomic Force Microscopy (AFM), which is suitable for investigating surfaces (all types of solid catalysts). It has a lower resolution (about 30 nm) than TEM and STM, but the apparatus is significantly cheaper. A higher resolution (down to 0.1 nm) can be reached by convolution techniques.
- Chemisorption of H_2_ or CO to determine the specific surface area of metallic crystallites/agglomerates (if the internal volume is accessible—see accessibility).
- Temperature Programmed Reduction (TPR) to determine/estimate the oxidation state of metallic or reducible particles.
- Temperature-Programmed Oxidation (TPO) to determine/estimate the valence state of a metal and the possibility of oxidizability.
- X-ray photoelectron spectroscopy (XPS) to determine the valence state of metal particles on the surface.

For metal catalysts, X-ray powder diffraction (XRPD) analysis, a rather cheap technique, holds a strong position. Besides estimating average crystallite size, it can also give some information about the support, similarly to zeolites [59], or layered hydroxides [475,476,477]. XRPD is also useful for verifying MOF structure after synthesis and modification [296,297,328,412,478,479,480,481] as well as phthalocyanine materials [287,482,483] and others [187,189,484].

Of course, the aforementioned list of characterization methods does not cover all techniques. Extended X-ray absorption fine structure (EXAFS) [485], X-ray absorption near edge structure (XANES) [486], and small-angle neutron scattering (SANS) [487] are good examples of more sophisticated methods for the characterization of texture. Besides the characterization of polymer samples in static mode, the investigation of dynamics during adsorption and desorption, e.g., water or CO_2_, is of great interest. Together with the evaluation of dynamic parameters (rate of adsorption, swelling/breathing of the adsorbent), the lifetime of the adsorbent can also be estimated [488].

For all catalysts, temperature and chemical stability are of great importance, which can be evaluated as follows:

- Thermogravimetric analysis (TGA)—the extent of decomposition of the carrier or catalyst with increasing temperature is measured.
- TGA in an oxidative atmosphere, which resembles TPO; however, the weight of the sample is monitored.
- TGA in a reductive atmosphere, which it resembles TPR; however, the weight of the sample is monitored.
- Differential scanning calorimetry (DSC)—decomposition and temperature effects are measured, e.g., release of water from the crystalline lattice and pyrolysis effects in organic polymer carriers.
- DSC followed by the analysis of degradation products (GC, GC, MS).
- (Micro)pyrolysis combined with pyrolysis product analysis (similar to DSC with analysis, other conditions).
- Hydrolytic, aminolytic, and other stability tests (these are of great importance for polymer-based catalysts).

Catalytic activity and selectivity are the last group of important tests, which include the following:

- Batch Reactor Tests (BRTs).
- Flow Reactor Tests (FRTs).
- Long-Term Tests (LTTs).
- Catalyst recycling in batch tests.
- LTT in flow-through reactors to observe activity reduction (reduction of key component conversion) and selectivity change.
- Tests under extreme conditions (long time, higher temperature, and/or pressure).

Generally, the presentation of systematic catalytic tests is rather rare in the available literature. One can tell that successful results can be the subject of a patent application rather than for publication. However, without assessing catalyst productivity and the costs associated with product isolation—factors that are heavily influenced by selectivity—it is nearly impossible to evaluate the quality of a catalyst accurately.

## 5. Examples of Applications

Most examples in the literature focus on batch reactors, with only a few addressing continuous reactors [80,203,408,449,489,490,491,492]. Below are a few representative examples of OMOP catalysts in industrial applications.

### 5.1. Esterification and Etherification

**Scheme 7:**
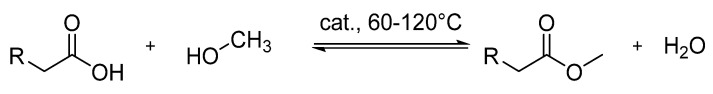
Esterification of fatty acids with methanol. Catalysts can be homogeneous (e.g., KOH, Ca((OH)_2_, H_2_SO_4_), or heterogeneous (e.g., alkaline oxides, SPSDVB).

Esterification and etherification are primarily carried out using acid catalysts; classically mineral acids [493]. The intention to immobilize acids on a polymer and use them as catalysts dates to the mid-20th century [494,495]. The focus and industrial applications are centered on sulfonated poly(styrene-co-divinylbenzene) [496,497,498]. A comparison of various acid catalysts, including Amberlyst-15 (25% crosslinked), XN-1010 (85% crosslinked), Nafion H, and K2661/K2631 (14–25% crosslinked) showed that the monodisperse K2661 catalyst was the most effective for the preparation of 1-phenylethyl acetate from acetic acid and styrene at 40 °C [442], even with a better selectivity compared with homogeneous catalysts (98% H_2_SO_4_, or *p*-toluenesulfonic acid). Polymer catalysts are also used for the esterification of fatty acids with methanol (Scheme 7) [499,500], which is crucial for producing engine fuels from renewable resources. Generally, acid resin-based catalysts for esterification and etherification are among the most successful, largely because of the relatively low temperatures at which these processes are conducted, typically ranging from 40 to 110 °C.

### 5.2. Alkylation

**Scheme 8:**
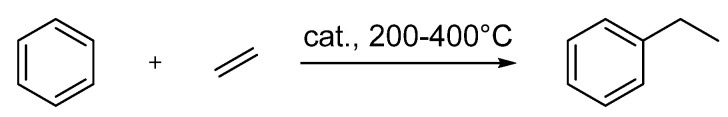
Alkylation of benzene with ethylene. Temperature depends on the type of catalyst (liquid phase with Lewis acids—lower temperature, zeolites—higher temperature).

Alkylation reactions using alkenes with acid-based catalysts are typically carried out at higher temperatures than esterification or etherification, ranging from 150 to 300 °C. For these temperatures, inorganic acids, such as zeolites, are more suitable [50,61,493]. Recently, Friedel–Crafts alkylations (such as the alkylation of benzene with ethylene, Scheme 8) have been performed using MOFs similar to zeolites [501,502]. These MOF catalysts exhibit long-term stability at 200 °C. However, there are concerns about the regeneration of MOFs compared with zeolites, which raises questions about whether MOFs can compete effectively with zeolites.

### 5.3. Hydrogenation

Hydrogenation reactions are commonly used to test metal-supported catalysts; cyclohexene and nitro compounds are most frequently employed as substrates [55,76,101,423,461]. When these processes are conducted at temperatures below 70 °C, catalysts generally remain stable. However, issues such as deactivation due to leaching in reactions with mesocompounds [55,103,503] and sintering of palladium particles [466] have been observed.

**Scheme 9:**
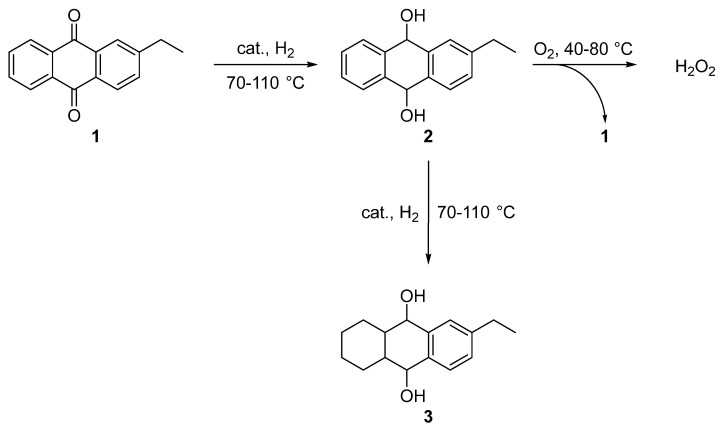
A simplified scheme of the synthesis of hydrogen peroxide (**1**—2-ethylanthraquinone, **2**—2-ethylanthrahydroquinone, **3**—tetrahydro-2-ethylanthraquinone (undesired side product, increases consumption of H_2_ and O_2_)).

Another important hydrogenation process is the reduction of substituted anthraquinones [57,504,505], which is a step in the production of hydrogen peroxide (Scheme 9). Despite significant efforts, OMOP catalysts have not demonstrated better economic performance compared with commercially used Pd/C catalysts [493].

Other hydrogenation reactions include the partial hydrogenation of benzene to cyclohexene [102], the hydrogenation of *α,β*-unsaturated aldehydes to produce unsaturated alcohols, the hydrogenation of oxygen in water for use in boilers, and the reduction of nitrates in water [57,100]. However, none of these processes have been scaled up to an industrial level.

Recent trends addressing the greenhouse effect and reducing fossil hydrocarbon use focus on the exploitation of CO_2_ through reactions with hydrogen and other reactants. More details about this topic are in Section 5.6 of this paper.

### 5.4. Oxidation

Successful oxidation processes using OMOP are rather rare because oxidation agents (such as O_2_, H_2_O_2_) can also attack the polymer structure. The extent of this attack—affecting the stability of the polymer network—increases with the activity and concentration of the oxidant and temperature. However, various components present in the reaction mixture—such as water, amines, and acids resulting from oxidation—can also contribute to the degradation of materials. For instance, while simple MOFs exhibit stability in neutral water, they tend to decompose under acidic or alkaline conditions [389]. To enhance resistance against acids, the use of carboxylate linkers and high-valent metal ions is beneficial. An example is MOF-545, which consists of [Zr_6_(µ_3_-O)_4_(µ_3_-OH)_4_(OH)_4_(H_2_O)_4_(COO)_8_ clusters synthesized from Zr^4^⁺ and tetrakis(4-carboxyphenyl)porphyrin (TCPP). Despite these improvements, their stability in alkaline environments remains only moderate.

**Scheme 10:**
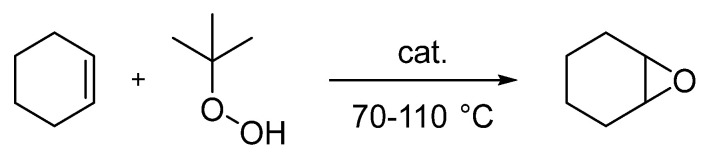
Oxidation of cyclohexene.

More stable polymers of benzimidazole, imides, phthalocyanine, and special MOFs have proved significantly higher stability than poly(styrene-co-divinylbenzene) and acrylamide supports. For instance, polybenzimidazole (PBI) resin-supported complexes of Cu, Mn, Fe, Ru, and Ti showed good activity and selectivity in cyclohexene oxidation using *tert*-butyl hydroperoxide (TBHP) and O_2_ at 60 °C (Scheme 10) [175]. In the liquid-phase epoxidation of higher olefins, a molybdenum(VI) complex supported on functional polyimide particulates with triazole groups achieved yields of oxiranes ranging from 70% (for styrene) to nearly 100% (for cyclohexene) at 60 °C or 80 °C [506]. This polyimide-supported Mo catalyst was highly active and selective and could be recycled 10 times with no detectable loss of Mo from the support.

Supported Cu(II) metal–porphyrins [284] were applied for dialkylphenol oxidative polymerization. Similar oxidation stability was also proved by Nafion-supported metal complexes in the oxidation of alkenes to oxiranes [284]. Functionalized commercially available epoxy resins bearing molybdenum polyoxometalates [191] exhibited excellent stability in the epoxidation of cyclohexene; these catalysts were used batchwise in up to 60 reactions over a period of 50 days, with an expected catalyst lifetime of approximately 1500 days and virtually no loss of activity. The selective oxidation of cyclooctene to epoxide was studied over Cu-containing MOFs at 75 °C in liquid toluene [507]. Additional results from other oxidation tests using various catalysts can be found in [416,508,509,510,511,512,513,514,515].

**Scheme 11:**
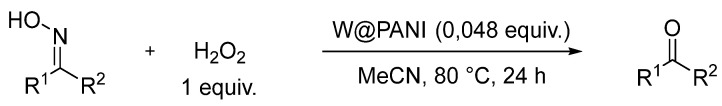
Deoximation reaction.

Interesting results from deoximation reactions (Scheme 11) using tungsten catalysts supported on polyaniline (PANI) (W@PANI) were reported in reference [516]. Yields of the corresponding ketones and aldehydes varied from 42% to 89% when benzaldoxime and acetophenone oxime were used as reactants, respectively (0.5 mmol of reactant, 20 mg of W@PANI, 0.5 mmol of H_2_O_2_, and 1 mL of acetonitrile at 80 °C for 24 h). The catalytic performance, measured as Turnover Frequency (TON), ranged from 0.8 × 10³ for benzaldoxime to 1.7 × 10³ for other reactants. A notable stabilizing effect was observed because of the O_2_WO group bound to the nitrogen in the PANI backbone, resulting in minimal metal leaching—approximately 0.06 ppm on average across tests with 23 different oximes—and confirming the reusability of the catalyst for at least two cycles. Additionally, the choice of solvent played a crucial role in these reactions. Acetonitrile (MeCN), a highly polar organic solvent with rich electron density in its π-system, exhibits a strong affinity for PANI, allowing the polymer backbone to swell. This swelling enhances the accessibility of the catalyst’s interior spaces. Furthermore, MeCN effectively dissolves both the oxime reactant and the oxidant (H_2_O_2_), creating an optimal environment for the catalytic reaction.

A specific challenge in organic technology is the availability of hydrogen peroxide, which is considered a very clean and green reactant [493]. Although the anthraquinone route remains the most important method for its production, direct oxidation processes for hydrogen are gaining increasing interest [517]. Various OMOP catalysts have been prepared and tested. Simple Pd catalysts supported on PBI and PVP showed virtually no activity in reactions involving oxygen, CO, and water [518]. Interesting results were obtained with Pd-Pt and Pd-Au catalysts supported on a strong cationic resin (sulfonated poly(styrene-co-divinylbenzene; commercial K2621 and K2622 samples) using CO_2_ as an inert gas [519]. A selectivity of H_2_O_2_ formation up to 43% with respect to hydrogen was achieved. A strongly acidic resin (Lewatit K2621) was also used in research [520]; a productivity of 1770 mol_H2O2_/mol_Pd_ h at 60% conversion of H_2_ and a selectivity of 57% to H_2_O_2_ were presented. The positive effect of acetonitrile as a solvent was reported in [521]. Comparisons of various sulfonated polymer supports with Pd/C catalysts [436] highlighted macroporous polydivinylbenzene (PDVB) as the most effective support for Pd crystallites. This can be attributed to a high specific surface (>700 m^2^ g^−1^) reached by the swelling of pristine PDVB in 1,2-dichloroethane (DCE). Subsequent sulfonation with concentrated H_2_SO_4_ charging with palladium acetate and reduction with H_2_ yielded a Pd particle size of about 10 nm (1 wt.% of Pd). The highest conversion of H_2_ was reached over non-sulfonated PDVB; however, the selectivity was very low (close to zero), indicating the importance of acidic sites. The best performance was observed with strongly sulfonated PDVP in DCE; yielding about 0.6 mmol H_2_O_2_/g_cat_, which was 6 times higher than over Amberlite IR 120- or Lewatit K2621-supported Pd (1 wt.% Pd) catalysts. However, it is important to note that even the most effective polymer-supported metal catalysts are only about half as productive in terms of H_2_O_2_ yield compared with Au-Pd catalysts supported on zeolites (2.5 wt.% Au and 2.5 wt.% Pd over zeolite Y) [522].

### 5.5. Coupling

**Scheme 12:**
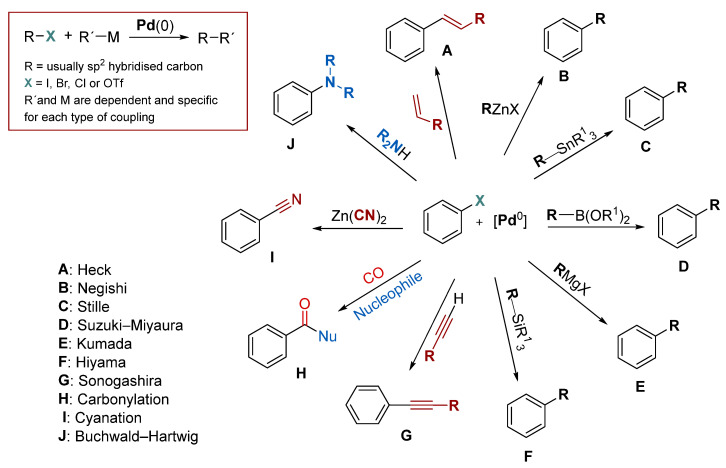
Various types of Pd-catalyzed coupling reactions.

Scheme 12 outlines various types of coupling reactions, with palladium species being the most frequently used catalysts for these processes [523].

Suzuki–Miyaura coupling (SMC) was performed over a chitin-modified supported palladium catalyst, with the catalyst demonstrating successful recycling up to five times. However, the yield gradually decreased from 94 to 81% (reaction conditions: bromobenzene (0.5 mmol), phenylboronic acid (0.75 mmol), K_2_CO_3_ (1.0 mmol), OCMCS-SB-Pd(II) (0.46 mol% Pd), and EtOH/H_2_O = 3:2, (5 mL) at 50 °C) [524]. The positive influence of microwave irradiation on SMC using palladium supported on chitosan is reported in [525]. Additionally, the beneficial effect of the heterogenization of a palladium complex on the bipyridine-based poly(arylene ether) is described in [526]; however, with increasing bulkiness of the reactants, the yields with heterogeneous catalysts were lower compared with the homogeneous catalytic process. Other successful supports for Pd catalysts in SMC include modified starch [214], modified MOFs [394], and polyimide [527].

Stille coupling (StC) is not so popular (probably because of the toxicity of organotin compounds) as SMC. Coupling between Sn(CH_3_)_4_ or *n*Bu_3_SnPh with iodoarenes or activated bromoarenes over palladium supported on a copolymer of 2-(acetoacetoxy)ethyl methacrylate with ethyl methacrylate and ethylene glycol dimethacrylate is reported in [528]. Yields higher than 80% (close to 100% in some cases) were achieved using 0.50 mmol of aryl iodides, 0.55 mmol of organotin compound, and 0.01 mmol of Pd (loaded on a polymeric support) in 2.0 mL DMF at 70 °C.

Negishi coupling (NeC) is a powerful process for the formation of C−C bonds. It involves sp3 carbons activated by organozinc reagents. Usually, NeC is performed as a homogeneous process with a palladium complex catalyst [529,530].

An OMOP core–shell catalyst for Kumada coupling (KuC) has shown notable effectiveness [531]. Reactions performed on the 0.80 mmol scale (arylhalide, (1 equiv)) with a second reactant (Grignard reagent, (1.2 equiv)), catalyst (5 mmol%), and THF (4 mL) at 0 °C to room temperature overnight provided desired products in 42–81% yield.

The PS-PdONPs catalyst, prepared by heating a mixture of Pd(OAc)_2_ and standard polystyrene in a 1.5 mol·L⁻¹ K_2_CO_3_ aqueous solution at 90 °C for 5 h, was successfully utilized in Hiyama coupling (HiC) [532]. This catalyst enabled the preparation of various substituted biphenyls using aryl bromides and trimethoxy(phenyl)silane at 80 °C for 3h in aqueous solution.

Sonogashira coupling (SoC) with PSDVB as a support for a palladium catalyst was successfully achieved using various substituted alkynes as nucleophiles [129]. Moreover, challenging carbonylative SoC was accomplished using Pd nanoparticles immobilized on metal scavengers (Smopex^®^-234, Smopex^®^-111, PVPy, 1 wt.%), yielding alkynyl ketones [142].

Heck coupling (HeC), also called the Mizoroki–Heck reaction, is the chemical reaction between an unsaturated halide (triflate) and an activated alkene in the presence of a base and a palladium catalyst, resulting in the formation of a substituted alkene. Because of its relative simplicity and safety, Heck coupling has been extensively studied [141,156,222,223,224,533,534,535,536,537,538,539,540,541,542,543,544,545,546,547]. Research has shown that various polymers have been employed to stabilize palladium particles in this process. However, the use of chloride compounds in the Heck reaction often leads to significant palladium leaching from the supports, primarily because of the formation of a stable PdCl_4_^2^⁻ complex.

Buchwald–Hartwig Coupling (BHC) is rather “young” (introduced in 1995). BHC is the metal-catalyzed synthesis of aryl amines from aryl halides or pseudohalides (for example, triflates) and primary or secondary amines. The utilization of Pd FibreCat (di(acetato)dicyclohexylphenylphosphinepalladium(II) bounded on polymeric FibreCat support) in BHC was described in [548]. The catalyst exhibited very good activity and selectivity; however, a significant leaching of palladium species was monitored.

Cyanation coupling (CyC), first developed in 1927, involves the reaction of a halide with a cyanide source (such as KCN, NaCN, CuCN, or Zn(CN)_2_) to produce organic cyanides. Because of the high toxicity of cyanides, the CyC reaction is exploited rather rarely. However, a modern approach to synthesizing benzonitriles often involves the cyanation of simple arenes, which do not possess a directing group. A complex view of CyC catalyzed by metal and non-metal components is provided in [549]. Similar to chlorine reagents, cyanides are able to form stable complexes, which contributes to the leaching of metals from the catalyst support.

Carbonylation and especially aminocarbonylation are of great importance for the synthesis of chemical specialties [112,113,550,551,552,553,554,555,556,557].

Recently, we reported the aminocarbonylation over palladium supported on polyureas [112,113]. Along with achieving good catalytic results in terms of activity and selectivity, the degradation of the support was also monitored. However, the catalyst developed in our laboratories proved to be more stable in comparison with commercially available (EnCat from REAXA).

### 5.6. CO_2_ Exploitation

The direct correlation between rising CO_2_ levels in the atmosphere and increasing global temperatures underscores the urgent need to reduce atmospheric CO_2_ (426 ppm as of July 2024, with a temperature rise of 1.17 °C since 1950) [558]. Reducing CO_2_ concentrations can be achieved through the following four primary strategies: (i) minimizing the consumption of energy, (ii) lowering the exploitation of fossil carbon sources (coal, crude oil, natural gas), (iii) introducing new low-carbon technologies, e.g., using green hydrogen instead of coal in metallurgy, and (iv) gradually removing CO_2_ from the atmosphere. These approaches, combined with a greater emphasis on bioresource utilization and a circular economy—promoting higher quality products with extended lifespans, effective recycling of used products, and responsible waste management [559]—can support a sustainable future on the earth. For a comprehensive review of CO_2_ capture and treatment methods, see reference [560].

The first step in CO_2_ sequestration or utilization in chemical transformations is to concentrate CO_2_ to over 90%, significantly higher than the typical concentrations found in flue gas (approximately 10–18 vol.%) or the atmosphere (about 0.04 vol.%). Industrial technologies often employ alkaline solutions, such as amines or alkali hydroxides, for CO_2_ capture [561]. For example, the “1PointFive technology” used in Direct Air CO_2_ Capture (DAC) employs a KOH aqueous solution to capture CO_2_. This process involves precipitating the formed K_2_CO_3_ with Ca(OH)_2_, which is derived from CaO and water, and ultimately generating pure CO_2_ through the thermal decomposition of CaCO_3_. Both KOH and CaO are recycled during the process [561,562]. While this method offers the advantage of producing a high-purity CO_2_ stream, it also comes with a relatively high energy requirement.

To achieve effective CO_2_ pre-concentration and separation, a solid adsorbent must possess high capacity, selectivity for CO_2_, rapid desorption, and low energy requirements for reactivation. A wealth of research exists on suitable inorganic (primarily zeolitic) and organic polymer adsorbents [298,563,564,565,566,567,568,569,570,571,572,573,574,575,576]. Notably, Zeolite 13X has demonstrated a maximum adsorption capacity of approximately 6 mmol/g [574], while a nitrogen-containing polymer, NUT-4 with an imine linker, has achieved 6.9 mmol/g [564]. Recently, the Zn MOF CALF-20, utilizing oxalate as a linker, was developed and successfully tested for CO_2_ separation [577,578,579,580,581,582]. BASF adopted CALF-20 technology and is collaborating with Svante to produce this MOF [583]. The process details include adsorption at around 90 °C and desorption using hot water steam at 120 °C, followed by drying and cooling to 60 °C. Each cycle lasts approximately 2 h (7900 seconds), and long-term tests indicate a lifespan of about 2 years for this adsorbent. Importantly, the adsorption mechanism involves van der Waals and covalent chemisorption of CO_2_ to the Zn atoms in CALF-20, providing both efficiency and selectivity for CO_2_ capture over N_2_, O_2_, and H_2_O, with relatively easy desorption facilitated by water steam.

Utilizing CO_2_ in chemical reactions is more complex than working with CO, as discussed in the previous section. CO_2_ is significantly more thermodynamically stable, with a Gibbs free energy of formation (Δ_f_G_298K_) of −94.26 kcal/mol, compared with −32.81 kcal/mol for CO [584]. When CO_2_ is concentrated, there are two main avenues for its transformation as follows: it can either be converted to CO using green hydrogen or directly utilized in reactions with excess hydrogen, such as in Fischer–Tropsch synthesis for generating alkanes and alkenes [493]. It is important to note that OMOP catalysts are not suitable for these processes, as they typically operate at temperatures exceeding 300 °C [585], which would likely degrade OMOP catalysts.

Fortunately, many processes can be conducted at temperatures below 120 °C. For example, modified MOF UiO-66 (see Figure 21) has been successfully used to convert CO_2_ into alkynyl carboxylic acids and cyclic carbonates, achieving yields exceeding 90% [586]. Research [410] has also reported the preparation of bis-cyclic carbonates through the addition of CO_2_ to epoxides at 30 bar and 120 °C, with the best results obtained using Al-OH–fumarate, which achieved 78.6% conversion and a turnover number (TON) of 1304 when carbonating 1,3-butadiene diepoxide, though stability data for the catalyst was not provided. Mesoporous melamine–formaldehyde resins have been employed in the heterogeneous continuous synthesis of cyclic carbonates from epoxides and gaseous CO_2_, specifically with epichlorohydrin, 1,2-butylene oxide, and styrene oxide. These processes achieved conversions and selectivities greater than 90% at temperatures between 120 and 140 °C and pressures of 20–50 bar; however, significant deactivation was observed after four recycling cycles [153]. Challenges in the direct reduction of CO_2_ to methanol using Zr6-MOF-based composite catalysts are discussed in reference [587]. While the research offers valuable mechanistic insights, it lacks quantitative data on catalyst recycling, which is crucial for process development. Additionally, a copper-functionalized zirconium MOF, termed Zr-CPB-Cu (CPB = 1,2,3,4,5,6-hexakis(4-carboxyphenyl)-benzene), demonstrated impressive results in the direct synthesis of styrene carbonate from styrene and CO_2_. This catalyst achieved a 97% conversion of styrene and a 92% yield of styrene carbonate under mild conditions (1 atm of CO_2_, 80 °C, and 12 h) using anhydrous tert-butyl hydroperoxide (TBHP) as an oxidant. Notably, Zr-CPB-Cu exhibited superior catalytic activity compared with the parent Zr-CPB and other MOFs, maintaining high activity after up to six reuse cycles [588].

Electrocatalytic reduction of CO_2_ (CO_2_ERR) offers the advantage of operating at lower temperatures compared with chemically catalyzed systems, but it does have the drawback of consuming electricity [246,248,407,589,590,591]. Commonly employed materials include metal complexes of phthalocyanine, metal-doped covalent organic frameworks (COFs), and metal–organic frameworks (MOFs). A comprehensive analysis of current advancements and future prospects can be found in reference [592]. Typical products of CO_2_ERR include carbon monoxide (often an undesired byproduct), methane, ethane, and various oxygenated compounds such as C1–C3 aldehydes, alcohols, acids, esters, and ketones. Despite the extensive research on CO_2_ERR, effective technological solutions remain elusive. A significant challenge is the deactivation of most organometallic organic polymer (OMOP) catalysts by water and organic reaction products. For instance, in contrast to fuel cells that operate on oxygen and hydrogen (which utilize Nafion membranes), organic compounds in CO_2_ERR systems—particularly acidic species—can severely degrade OMOP catalysts. A key reason for this deactivation is the strong chemisorption of organic acids on metal atoms, similar to what occurs in pure inorganic catalytic systems. This chemisorption is reversible; however, maintaining a high ratio of H_2_ to CO (often exceeding 15) can help mitigate the issue by adjusting the electric potential, typically changes within the range of 0.2 to 0.5 V [593]. As emphasized in the referenced studies, ongoing research and long-term experiments are essential to advance this technology.

Photocatalysis using sunlight offers lower operational costs compared with electrochemistry, yet the productivity of these systems tends to be relatively low, typically measured in grams of product per gram of catalyst per hour (g/g_cat_/h), and highly dependent on sunlight intensity [251,255,379,594]. An insightful overview [595] discusses various organometallic organic polymer (OMOP) photocatalysts, such as Au₁₉@ZIF-67 and Ni_0_._75_Mg_0_._25_-MOF-74, alongside inorganic photocatalysts like Pt/In_2_O_3_/g-C_3_N_4_, *µ*-CoAl-LDH, and pg-C_3_N_4_/Ti_3_AlC_2_/TiO_2_. The paper highlights the potential of integrating different photocatalytic approaches, including UV-Vis light conversion for OMOP photocatalysts, light-induced photon-thermal conversion for inorganic photothermal catalysts, and high-temperature solar conversion for thermal inorganic catalysts. However, further research is essential to explore and optimize these technologies fully.

## 6. Industrial Large-Scale Applications

Despite the versatility of OMOP catalysts, their large-scale applications remain limited. For instance, processes like the one-step synthesis of methyl isobutyl ketone (MIBK, 4-methylpentan-2-one) [596] have proven to be less economical compared with traditional two- or three-stage methods (acetone coupling to form diacetone alcohol (DAA) catalyzed by a base, dehydration of DAA to mesityl oxide (MOX) using an acid catalyst, and hydrogenation of MOX to MIBK over a redox catalyst [493]. Similarly, the removal of oxygen from water for boilers using hydrogenation over a Pd-supported catalysts is safer to perform using hydrazine or hydrogen over a metal catalyst dispersed on alumina. The outlined economic and environmental aspects excluded the abovementioned processes from large-scale applications. However, acid-catalyzed processes are examples of successful industrial applications of functionalized resin-based catalysts (“heterogenized homogeneous catalysts”).

### 6.1. Alkyl-Tert-Butyl Ethers

**Scheme 13:**
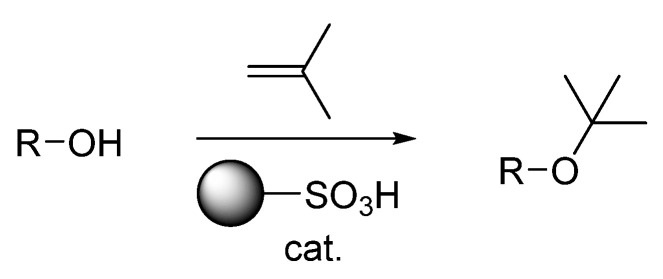
Synthesis of alkyl *tert*-butyl ether from isobutene and alcohol.

Alkyl *tert*-butyl ethers (Scheme 13) are important components of engine fuels (increase in octane number, reduction of toxic side products during incineration).

Initially, from 1980 to 2010, methyl *tert*-butyl ether (MTBE) was produced using microporous sulfonated poly(styrene-co-divinylbenzene) (SPSDVB) [497]. This process employed catalytic distillation, operating at temperatures below 80 °C, to prevent catalyst deactivation from alcoholysis. However, around the year 2000, the production of MTBE was phased out because of its high water solubility and volatility, which raised concerns about toxicity. This led to the adoption of ethyl *tert*-butyl ether (ETBE) as an alternative [597]. ETBE production also utilizes reactive distillation over SPSDVB but operates at a slightly higher temperature of approximately 100 °C, which is still manageable for maintaining catalyst longevity over several months.

Efforts to develop more efficient catalysts for etherification, such as sulfonated Nafions and regenerable zeolites [498], have not yielded significant improvements.

The annual world production of MTBE and ETBE is about 35 million tons. ETBE technology has underlined its importance because of possibility of using bioethanol and preparing isobutene from it. So, ETBE as a fully renewable product with the name Bio-ETBE has been introduced.

### 6.2. Bisphenol A

**Scheme 14:**
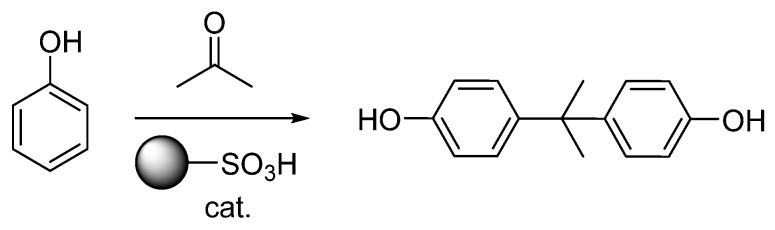
Synthesis of Bisphenol A.

Bisphenol A (BPA) (Scheme 14), despite its hazardous properties [598,599], continues to be a crucial component in the production of epoxides. Its synthesis is typically carried out using macroporous SPSDVB [600]. Because of the rather bulky molecule of BPA and low polarity of reactants, optimizing the texture of SPSDVB and reaction conditions was necessary [601,602,603]. In 2022, the global production of BPA was approximately 10 million tons, with an anticipated increase in production in the coming years.

## 7. Deactivation

**Figure 23:**
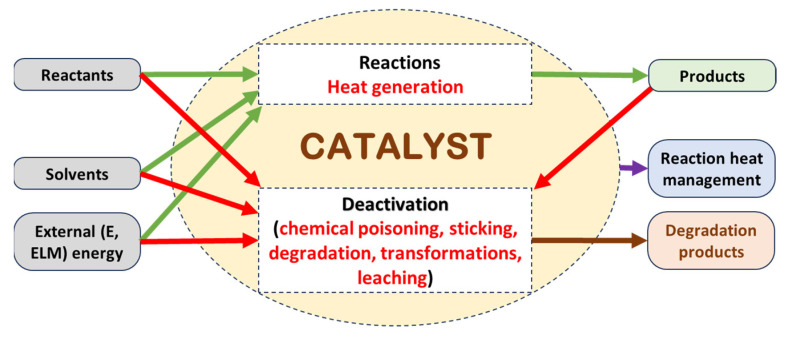
Deactivation of catalysts (E–electric energy, ELM—electromagnetic energy).

The deactivation of heterogeneous catalysts poses significant economic and ecological challenges in processes utilizing solid catalysts [604] (Figure 23). The main processes in deactivation are as follows:

(i) Chemical poisoning, e.g., sulfur moieties non-reversibly adsorbed (chemically bound) on metal particles.
(ii) The sticking of side products on the catalyst surface (also called fouling).
(iii) Degradation, which can be mechanical (abrasion), in stirred systems, or by the chemical action of species in a reaction mixture, the splitting of catalytic active parts (e.g., sulfonic group, amino group, metal–organic complexes) can be also considered as a degradation process.
(iv) Transformation of catalytic active parts, e.g., sintration of metal nanoparticles leading to bigger ones with a lower surface and lower catalytic activity, or reaction with some components from the reaction mixture, e.g., oxidation by a nitro group in the hydrogenation and formation of some soluble products.

Degradation products from both the support material and the catalytic species can migrate into the reaction mixture, complicating separation processes and negatively impacting ecological systems. Addressing these issues is essential for improving catalyst longevity and sustainability.

When external energy sources (such as electricity, sonic, microwave, and light) are employed to accelerate the desired reactions, they can inadvertently enhance deactivation processes, primarily through increased temperature. Consequently, effective heat management during reactions becomes crucial. Reaction heat management can be ensured by cooling/heating or by the evaporation of volatile components from the reaction mixture, condensing evaporated components for recycling back into the system (reactive distillation). Implementing these heat management techniques can help optimize reaction efficiency while minimizing catalyst deactivation.

The degradation of supported organometallic organic polymer (OMOP) catalysts is significantly influenced by the interaction with components from the reaction mixture. It is noteworthy to consider the strength of the following chemical bonds (kJ mol^−1^) [605]: C-C (346), C-C in benzene (585), C_6_H_5_-CH_2_C_6_H_5_ (273), C_6_H_5_-C_6_H_5_ (478), H-C_6_H_5_ (465), C-S (272), and C-N (305). The weaker C-S and C-N bonds, in comparison with carbon bonds in aromatic rings, make them more susceptible to attack (and potential cleavage) by reaction components and/or solvents. This is particularly relevant for vinyl-type polymers (SPSDVB and APSDVB). In contrast, when nitrogen is incorporated into aromatic systems (such as aromatic polyamides—aramids, PBI, some COFs, and composites [392]), the presence of hydrogen bonds significantly enhances molecular stability [606,607]. As a result, these materials can endure harsher conditions than vinyl-type polymers.

Strong chemisorption of reaction species on the catalyst surface can resemble poisoning. The effects of this phenomenon can be minimized through a dynamic catalytic regime, such as sweeping voltage and/or varying the concentration of reactants in electrochemical CO_2_ treatment. This approach is also applicable in photocatalytic conversion. In this system, intermediate CO is formed and reacts with hydrogen on the catalyst surface. When the surface is blocked by strongly chemisorbed CO, the voltage is adjusted to a level where the reduction of CO_2_ occurs at a slower rate. This allows the chemisorbed CO to react with hydrogen, which in turn activates the catalytic surface. Once this reaction has taken place, the system can return to a faster regime, increasing the CO_2_ concentration and resuming the higher-voltage conditions required for rapid reactions.

For OMOP-based catalysts, the following groups need to be distinguished:

- Non-metal-containing polymer catalysts, e.g., SPSDVB.
- Catalysts containing metals as coordinated atoms and metal particles.

Non-metal-containing polymer catalysts are deactivated by the following:

- Splitting of functional groups (e.g., -SO_3_^−^, -NH_2_ from the polymer backbone).
- Splitting/scission of bonds in the polymer chain.
- Sticking of higher molecular side products on the body of the catalyst.

All these processes are accelerated by the “destruction capability” of the components of the reaction mixture and temperature. It is possible to suggest the following order of polymer resistance against degradation (mainly temperature, but also oxidative/reductive, hydrolytic, and aminolytic degradation):

- Linear polymers.
- Crosslinked polymers not containing aromatic rings.
- Crosslinked polymers with aromatic rings (e.g., PSDVB).
- Simple COFs.
- Polyanilines.
- Aromatic polyamides.
- Polyimidazoles and polybenzimidazoles.
- Modified COFs (not containing metals).

Of course, as for the deactivation by side products, it does not depend on the type of polymer.

An interesting study on deactivation during the synthesis of Bisphenol A (Scheme 13) is reported in [601]. A highly crosslinked SPSDVB was used in a continuous fixed-bed reactor, with ethyl mercaptan serving as a promoter. Laboratory experiments, mathematical modeling, and optimization of the production reactor’s regime enabled the catalyst to achieve a lifespan of over 1000 h. This extended lifespan significantly contributes to the economic efficiency of the process.

The deactivation of metal (atomic or nanoparticle)-containing catalysts depends on the character of the metal as follows:

◦ MOFs and metal-containing COFs can be deactivated by the collapse of the structure as follows:
  - Effect of temperature.
  - Scission of the linker bond with the metal by moieties of the reaction mixture.
◦ Catalysts with dispersed metals are also deactivated by the following:
  - Transformation of metal particles to soluble form, e.g., oxidation and complexation.
  - Sintering, even at relatively low temperatures (40 °C); see, e.g., [466].
  - Action of metal particles on the polymer network resulting in the splitting/scission of bonds on the polymer chain.

An excellent example of the deactivation and reactivation of a potassium catalyst is provided in reference [608]. Under optimal conditions, the hypercrosslinked polystyrene catalyst, prepared from styrene, benzaldehyde, and dimethoxymethane and charged with KOH (HCP-SB-K), achieved a yield of 99.9% for Fatty Acid Methyl Esters (FAMEs) at 60 °C over 2 h with 3% *w*/*w* catalyst. However, when using the recycled catalyst, the yield dropped to only 42.5% because of deactivation from fouling. After regeneration through impregnation with KNO_3_ and calcination, the catalyst exhibited a yield of 98.2% FAMEs, similar to that of the fresh catalyst. Evaluation of potassium leaching indicated no loss of basic sites.

For both MOFs and dispersed metal catalysts, the problem of sticking by higher molecular side products exists.

Our attention has focused on two aspects of deactivation, namely, the dissolution and leaching of metal, and the degradation of the polymer support [102,103,105,503]. The dramatic decrease in activity due to the destruction of the polymer support, in a selective hydrogenation of benzene to cyclohexene over ruthenium catalysts, carried out at 150 °C [102], forced us to use an inorganic zeolitic support instead of a functional organic polymer. As an interesting phenomenon, the redeposition of palladium and growth of metal particles in the body of polyurea support, which gradually degraded, was observed [112,113]. In aminocarbonylation experiments, a Pd (2 wt.%) catalyst degraded to about 40% of its weight with respect to the starting value after five reaction cycles. However, the content of palladium increased to about 5 wt.%, and the size of Pd crystallites increased from about 4 nm to 12 nm. This high redeposition extent of palladium was ensured by the strong chelating capability of urea and amino groups in the polyurea support functionalized with 2-(aminomethyl)pyridine. The fixation of palladium suppresses leaching to the reaction mixture, simplifying the treatment of the reaction mixture and the isolation of palladium from the used catalyst. All these features underline the advantage of using OMOP-based catalysts in aminocarbonylation systems.

The leaching of metals can be suppressed by the following:

- Lowering the activity of reactive components, e.g., hydrogenation of nitro compounds conducted at low concentration, optimally in a continuous system, in which the concentration is immediately decreased after entering the reactor. Such arrangement is established in industrial systems [493].
- Multimetal catalytic systems, e.g., Pd-Co catalysts in the hydrogenation of nitro compounds [503, 608], or special Pt-Pd-Fe for hydrogenation of nitrobenzene to aniline.
- Chelating ligands anchored to the support [112,113,523].
- Chemically bound catalytic moieties, e.g., in the deoximation over the tungsten catalyst [516].

The most relevant information about catalyst stability is revealed from catalytic tests. If batch experiments are carried out, at least two experiments with recycled catalysts should be performed, and in the case of metal-containing catalysts, the content of metal moieties in the reaction mixture must be established. In a continuous system, changes in the output composition at constant input parameters must be monitored.

## 8. Disposal of the Used Catalysts

In comparison with inorganic catalysts, e.g., treatment of used automobile catalytic converters [609], OMOP-based catalysts do not have such protocols. OMOP catalysts not containing metals can be treated as polymer waste, e.g., by incineration. However, the presence of heteroatoms, namely, sulfur, nitrogen, and phosphorus, has to be considered for incineration conditions and cleaning the flue gas.

In the case of metal-containing OMOPs, two possible routes include the following:

- Incineration, similar to the treatment of metal catalysts supported on carbon [610].
- Extract metals and then treat them as polymeric waste not containing metals.

As for extraction of metals, mineral acids, organic acids (e.g., oxalic), or chelating agents can be used.

## 9. Conclusions

Organic and metal–organic polymer (OMOP) catalysts have been among the most actively researched areas in the past 80 years.

The ease of preparing new catalytic materials with a well-defined structure and functionality (MOFs, COFs, core–shell catalysts, etc.) and diverse techniques suitable for characterization have resulted in sophisticated (rather expensive) catalysts. The availability of commercial more complex materials (encapsulated catalysts, COFs, MOFs, special linkers, etc.) and the possibility to exploit them gives the chance to perform more advanced catalytic processes. However, in most of the published research, recyclability, the catalyst’s lifetime, and the impact of lower selectivity on separation processes are usually not adequately considered, despite their economic implications. In contrast with previous statements, the recyclability of catalysts is often excluded in the synthesis of drugs. Every batch requires a new (well-defined) catalyst. Therefore, the price of an original catalyst plays a much more significant role than in the case when the recycling of the catalyst is possible.

From the findings presented in this article and detailed in the referenced papers, the following conclusions can be drawn regarding the catalytic applications of OMOP catalysts:

- Acid solid catalysts (SPSDVB): These catalysts have demonstrated significant potential for esterification, alkylation, and etherification reactions, including those at large industrial scales. When larger molecules are present in the reaction mixture, macroreticular catalysts are necessary. These are commercially available and can reliably operate at temperatures up to 120 °C.
- Basic-type catalysts: Catalysts containing amine groups and/or tetra-alkylammonium are generally less stable and not suitable for temperatures exceeding 100 °C; however, they are commercially available.
- Catalysts with more active functional groups: Catalysts with active groups, particularly those containing nitrogen (-CO-NH-) and amine groups, are well-suited for metal catalysts because of their strong chelating capabilities. Their commercial availability and ease of preparation position them as strong candidates for producing specialty chemicals via chemical coupling, carbonylation, and amino carbonylation reactions. Their combination with inorganic supports is also noteworthy.
- Bounding metal moieties: Binding metal moieties (e.g., -O-WO_2_) and complexes (primarily phosphine types) to less strongly functionalized polymer backbones present a challenge for successful applications, typically limited to temperatures up to 120 °C.
- CPs, COFs, and MOFs: Despite their widespread commercial availability, the applications of CPs, COFs, and MOFs in classical chemical catalysis are limited, likely because of their costs. In comparison with OMOPs, which have random polymer structures, COFs and MOFs feature more rigid architectures. This rigidity can hinder the accessibility of reaction species because of increased transport limitations. Smaller particle sizes (micron scale) may help mitigate these transport issues. Similar to zeolites, COFs and MOFs have well-defined porous structures, which can enhance selectivity for stereoselective reactions, such as those used in biologically active material synthesis. Operating temperatures should be kept below 150 °C. Notably, no commercial applications of CPs, COFs, or MOFs in chemical catalytic systems have been identified to date, although their adsorption capabilities—such as CALF-20 for CO_2_—are well-documented, leading to industrially developed processes. Their potential in electronics, analytics, and photocatalysis remains indisputable.
- Preparation of catalysts based on renewable materials: The use of catalysts derived from renewable materials (e.g., wood, cellulose, biochar, starch, chitin, chitosan) has been undervalued so far.

Recommendations for an economic application of OMOP catalysts in liquid phase catalytic processes (including those in which liquid is formed by adsorption and capillary condensation) include the following:

- For a target process, consider that OMOP-based catalysts are very often hybridized homogeneous catalysts; atypical example is SPSDVB. Immobilized metal complexes, or enzymes, are also heterogenized catalysts. It is strongly advised to perform a catalytic reaction with non-heterogenized catalysts and compare the activities.
- Deposition/anchoring of catalytic metal moieties close to the surface of the catalyst particle to minimize the amount of used metal and decrease the price of the catalyst.
- Assess the size of catalyst particles and the effects of mass transport on reaction rates.
- Dispersed metal catalysts resemble those supported on inorganic materials, yet OMOPs offer greater functionality, presenting a significant advantage. Inorganic supports can also be functionalized to introduce oxygen or nitrogen groups.
- OMOP catalysts can face significant deactivation issues, often losing stability over time. Understanding interactions with reaction components, the impact of temperature, and the overall catalyst lifespan is critical. Higher crosslinked polymers and fortified COFs and MOFs exhibit greater stability but may compromise interior accessibility. A balance must be sought between reaction rate and catalyst lifespan as follows:
  - For vinyl polymer-based OMOP, use a maximum of 120 °C.
  - For PBI, PU, and stabilized COFs and MOFs, use a maximum 150 °C.
  - When utilizing external energy sources (light, microwave, ultrasound), ensure that doses are controlled to prevent the overheating of OMOPs beyond their specified limits.
- A general rule of thumb is to begin with commercially available, cost-effective, and well-characterized catalysts when exploring a new process.
- When developing new catalysts, prioritize renewable materials (wood, cellulose, biochar, starch, chitin, chitosan). If they do not meet requirements, consider more complex synthetic functionalized polymers.
- For both commercial and newly prepared catalysts, reproducibility in their preparation and recyclability are crucial.
- Minimize the presence of species that can attack OMOP, particularly water, amines, acids, and other reactive compounds, e.g., by dilution of the reaction mixture with some inert solvent.
- When using oxygen or hydrogen as reactants, apply pressures that minimize attacks on the polymer backbone, ideally atmospheric or slightly elevated pressures (generally not exceeding 10 bar).
- Take into account that the degradation of OMOP increases much more with temperature in comparison with inorganic catalysts.
- In the case of metal-containing OMOPs, apply chelating agents anchored to the polymer backbone to minimize the leaching of metals.
- Minimize the activity (concentration) of reaction species capable of reacting with metal particles, or anchored complexes, e.g., if products have no such degradation capability, operate in a continuous stirred system, which allows for a quick decrease in the reactant concentration.
- Consider treatment and disposal of the used catalyst.
- If the chosen OMOP catalyst is not sufficiently stable under reaction conditions, transition to inorganic catalysts and investigate them by similar routes as described above.
- For specific products, compare the overall economics of homogeneous and heterogeneous catalytic processes, considering the following:
  ◦ Estimated/commercial prices of target product vs. the price of raw materials.
  ◦ Price of the catalyst.
  ◦ Catalytic reaction—reactor, energy, analysis, and human resources.
  ◦ Separation—equipment, energy, analysis, and human resources.
  ◦ Regeneration “overhead expenses“.
- Decide between a homogeneous and heterogeneous process.

These recommendations aim to prevent scenarios where a newly designed and characterized OMOP catalyst performs exceptionally well initially but later loses its advantageous properties, akin to an “enfant terrible” in a relationship. To ensure an OMOP catalyst serves as a reliable “assistant,” its specific characteristics must be carefully considered.

Despite the vast number of references included in this paper (more than 600), not all peculiarities of OMOP catalysts were introduced. For example, imprint polymers, catalysts with chiral centers for stereoselective reactions, and other special topics. An overview of these materials can be an inspiration for the future.

## Data Availability

Data cited from the papers by the authors are available on request.
